# Electrostatic Preorganization in Three Distinct Heterogeneous Proteasome β-Subunits

**DOI:** 10.1021/acscatal.4c04964

**Published:** 2024-10-02

**Authors:** Silvia Ferrer, Vicent Moliner, Katarzyna Świderek

**Affiliations:** BioComp Group, Institute of Advanced Materials (INAM), Universitat Jaume I, Avenida de Vicent Sos Baynat, s/n, 12071 Castellón, Spain

**Keywords:** electrostatic effects, enzymatic catalysis, QM/MM, 20S proteasome, spatiotemporal theory

## Abstract

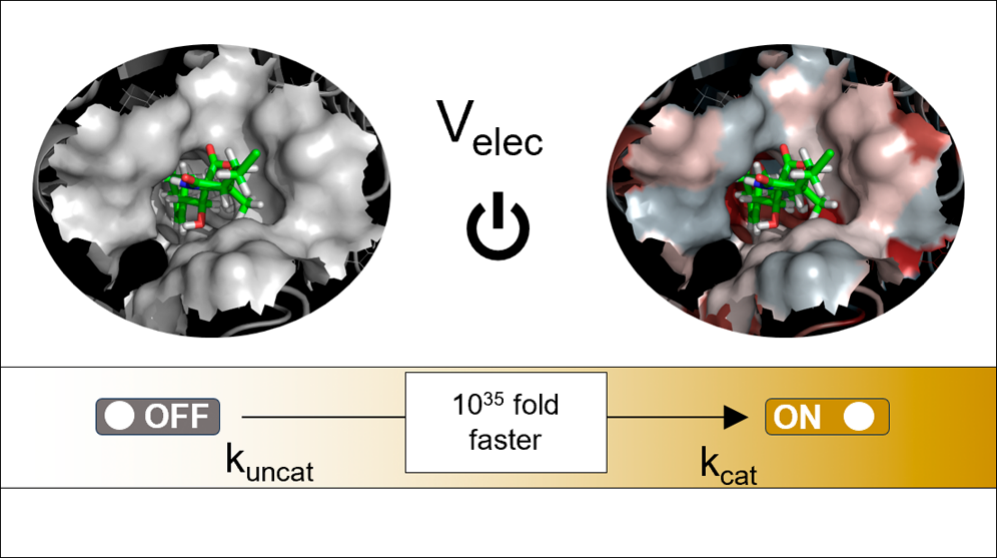

The origin of the enzyme’s powerful role in accelerating chemical reactions is one of the most critical and still widely discussed questions. It is already accepted that enzymes impose an electrostatic field onto their substrates by adopting complex three-dimensional structures; therefore, the preorganization of electric fields inside protein active sites has been proposed as a crucial contributor to catalytic mechanisms and rate constant enhancement. In this work, we focus on three catalytically active β-subunits of 20S proteasomes with low sequence identity (∼30%) whose active sites, although situated in an electrostatically miscellaneous environment, catalyze the same chemical reaction with similar catalytic efficiency. Our in silico experiments reproduce the experimentally observed equivalent reactivity of the three sites and show that obliteration of the electrostatic potential in all active sites would deprive the enzymes of their catalytic power by slowing down the chemical process by a factor of 10^35^. To regain enzymatic efficiency, besides catalytic Thr1 and Lys33 residues, the presence of aspartic acid in position 17 and an aqueous solvent is required, proving that the electrostatic potential generated by the remaining residues is insignificant for catalysis. Moreover, it was found that the gradual decay of atomic charges on Asp17 strongly correlates with the enzyme’s catalytic rate deterioration as well as with a change in the charge distributions due to introduced mutations. The computational procedure used and described here may help identify key residues for catalysis in other biomolecular systems and consequently may contribute to the process of designing enzyme-like synthetic catalysts.

## Introduction

Enzymes, the most efficient and environmentally friendly catalysts, have attracted large attention from researchers for decades due to the possibility of catalyzing new-to-nature chemical processes.^1^ For this purpose, hybrid molecular systems can be created that combine the broad reaction scope of synthetic catalysts with the exceptional catalytic performance, selectivity, and mild reaction conditions offered by protein scaffolds.^2,3^ Thus, many now recognize progress in enzyme design as a crucial step toward transitioning to a sustainable economy. Although significant work has been done in developing computational^4,5^ and experimental techniques^6^ allowing for protein modifications, the variety and efficiency of designed artificial enzymes are still minor, mostly due to limited knowledge about the origin of their catalytical power.

The origin of the enzyme’s role in accelerating chemical reactions is one of the compelling and still fundamental questions. The intense scientific debate about how enzymes participate in this phenomenon has been going on for more than seven decades. Over this period, several catalytic proposals were formulated. Still, most of them that were introduced to explain the catalytic effect of enzymes, such as desolvation,^7^ tunneling,^8,9^ covalent effects,^10^ compression effects,^11^ or dynamic effects^12,13^ were demonstrated to contribute not more than a few kilocalories per mol to the catalytic effect, compared with the counterpart reaction in solution, therefore could not be considered as the major reason for the increase in reaction rates.^14−19^ A similar conclusion was originally reached regarding electrostatic effects. First experiments with small compounds in solution^18,20^ which explored the role of electrostatic effects by introducing charged groups assumed to stabilize the transition state (TS) charge distribution, concluded that such effects must be small. Similarly, attempts to estimate the magnitude of electrostatic contributions to catalysis^21^ also indicated that such effects were negligible. However, because the conclusions elaborated based on data measured for small organic compounds in solution are not necessarily conclusive for enzymatic systems, a new insight into the influence of electrostatic effects on enzymatic catalysis was proposed in a study by Warshel and Levitt which provided the first quantitative proof that electrostatic effects can play a major role in this process.^22^ Their pioneer work together with many published afterward by themselves^15,23^ by us^16,17,24−27^ and others^28−36^ on various enzymatic models suggested the participation of the electrostatic field generated in the preorganized active site in the stabilization of the TS structure and, in consequence, in the acceleration of chemical transformation. The role of the Coulombic environment has been indirectly confirmed by using different variants of the same enzyme obtained by introducing specific mutations or by comparative studies of the same reaction processes taking place in an aqueous environment and the active site of the protein. The successful use of oriented external electric fields to accelerate the rate of chemical processes also supports the catalytic role of electrostatic effects.^37−40^ Most of these studies have been already summarized in several recently published reviews.^14,31,41^

The 20S proteasome, a large intracellular system, was chosen as an example to test the role of the electrostatic effects generated by the protein in enzymatic catalysis in the present study. From a biological point of view, the 20S proteasome is the primary protease for proteolysis in eukaryotes, and its role is to eliminate misfolded, damaged, or unneeded proteins and supply amino acids required for the synthesis of novel proteins.^42−44^

The proteasome participates in various cellular metabolic processes; therefore, its deregulation can cause many disorders, including neurodegenerative and autoimmune diseases.^45,46^ Moreover, it has also been validated as an anticancer drug target and has since become intensively studied for inhibitor design.^47,48^

**Figure 1:**
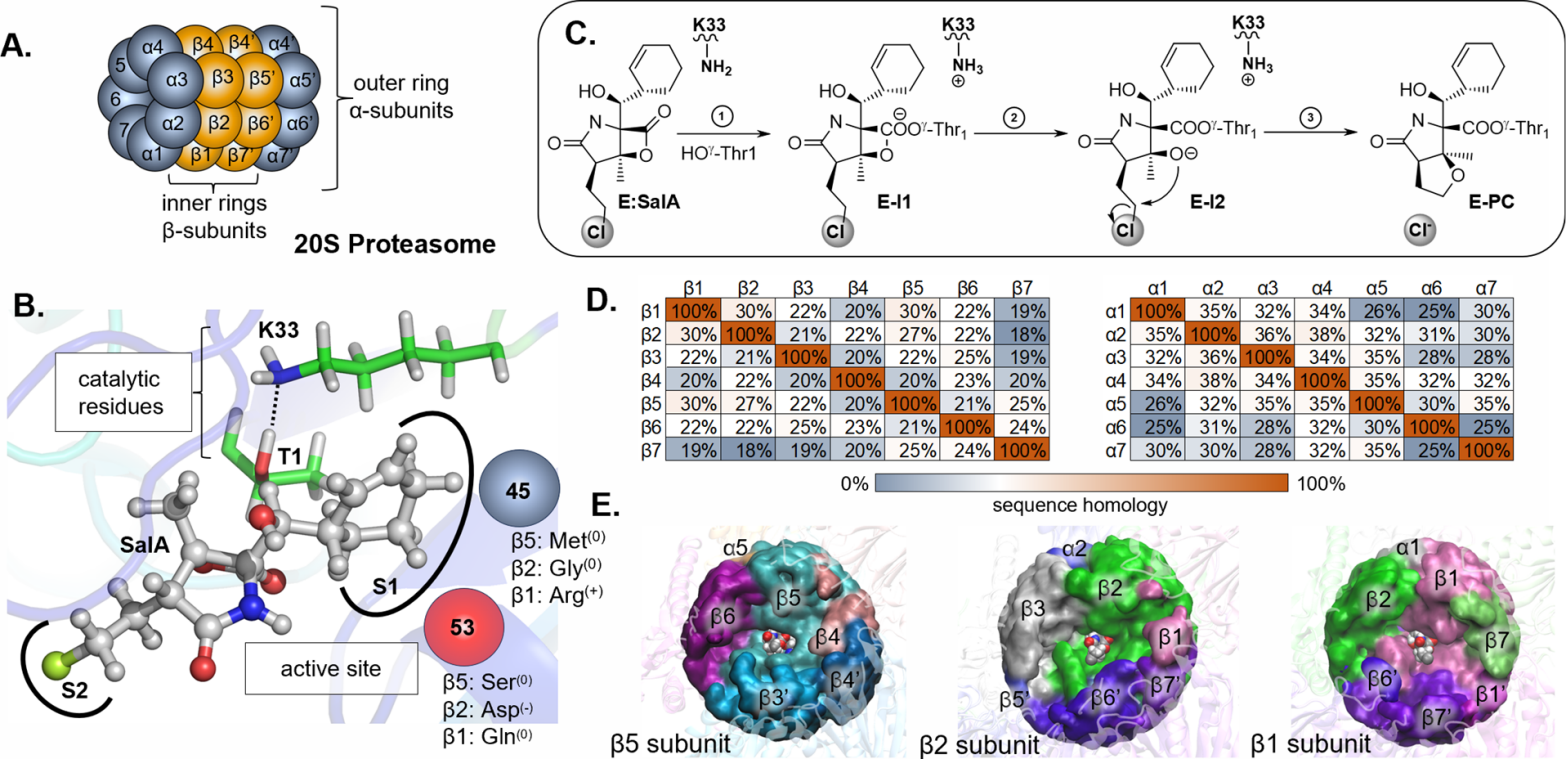
(A) Schematic representation of the 20S Proteasome with the relative positions of α1–7 and β1–7 subunits. (B) Active site of active β-subunits including the position of substrate, conserved catalytic residues, and S1 pocket amino acids essential for the recognition step. (C) Molecular mechanism of the covalent product formation between SalA and the active site of the β5 subunit, as revealed in the previous computational studies. (D) Relative identity of α and β subunits sequences. (E) Subunits located within a cutoff distance (20 Å) from the active site of each active β-subunits.

The 20S proteasome is a cylindrical particle composed of four axially stacked rings with two outer rings formed by seven different α-type subunits and two inner rings made of seven different β-type subunits, as shown in Figure 1A. Three of the β subunits of each β ring (β5, β2, and β1) are peptidases, and each has a distinctive specificity (chymotrypsin-like, trypsin-like, and caspase-like, respectively). β5, β2, and β1-subunits are N-terminal nucleophile hydrolases in which the N-terminal threonine (Thr1) plays the role of nucleophile in the hydrolytic reaction.^49,50^ Behind proteasome specificity lies the singularity of the S1 pocket architecture of each subunit. Thus, β1, characterized by the presence of positively charged Arg45 in S1, binds polypeptides with a basic side chain in position P1. β2 subunit consists of negatively charged Asp53 that favors binding of an acidic group of P1. Finally, neutral Met45 present in S1 of β5 generates its hydrophobic character,^51^ as shown in Figure 1B.

The main reason for using this particular molecular machine in our study is the presence of three unique active sites embedded in a complex protein matrix that despite significant structural composition differences catalyze the same chemical process. In the present study, we have selected the covalent inhibition by an acylation and intramolecular cyclization of Salinosporamide A (SalA), which has shown similar reactivity in the three different sites.^52^ Therefore, this chemical transformation in each of the catalytically active subunits was explored by employing computational chemistry techniques based on quantum mechanics/molecular mechanics (QM/MM) methods. The use of the neutral SalA compound in this study has an additional advantage because it allows avoidance of undesirable electrostatic effects originating in the presence of electrostatically dissimilar substituents (due to their complementarity toward the binding pocket) in the P1 position, which subsequently could camouflage or distort the research results and influence the final conclusions. SalA, as demonstrated experimentally and computationally, binds by adopting the same poses in all three active sites^25,53^ and as mentioned reacts with similar rate constants.^52^ The mechanism of the reaction in the β5 subunit was recently described based on a computational approach in our laboratory,^25^ demonstrating that the active site is inactivated by SalA after three chemical steps, as shown in Figure 1C. Importantly, lysine 33 was determined as a threonine (Thr1) activator, a function that was indicated by experimental results.^54−56^ and confirmed by other computational studies.^57−59^ In detail, the proposed reaction mechanism involves the abstraction of the proton from Thr1 by Lys33 accompanied by the nucleophilic attack of Thr1 on the carbonyl carbon of SalA of β-lactone, which is followed by β-lactone ring-opening. In the last step, the nucleophilic displacement of the side-chain chlorine atom by the alcoholate leads to the formation of the tetrahydrofuran (THF) ring, which is the final structure of the covalent adduct β5-SalA formation.

The purpose of this study was to examine why all three catalytically active β-subunits of the 20S proteasome identically catalyze the irreversible covalent binding of SalA and what is the electrostatic contribution of the protein residues in enhancing the chemical process. For this purpose, mechanisms of β2 and β1-subunit inactivation were explored and compared with one previously described for the β5.^25^ Then, the computed free energy (Δ*G*^‡^) barriers were confronted with the electrostatic potential (*V*_elec_) generated by the environment provided by each subunit and its surroundings in which chemical transformations take place. To clarify the role of the electrostatic field in catalysis, calculations for the reaction mechanism were repeated in the absence of point charges on the atoms constituting the protein and solvent. Because the prediction of the role of *V*_elec_ of each residue in the catalytic effect employing experimental techniques is not straightforward, we designed a computational procedure in which the contribution of individual amino acid residues to the electrostatic effect can be quantified.

## Results and Discussion

### Structural Analysis of Catalytic Subunits

Seven α-type(α1–7) and seven β-type (β1–7) subunits, building blocks of human 20S proteasome, are nonidentical proteins as reflected by poor sequence homology shown in Figures S1–S3 and 1D. Together they share only 17 common positions with identical amino acid residues in the case of α and only five in the case of all β-subunits. However, and interestingly, the secondary structures of all α-subunits as well as β-type subunits have almost identical folding patterns, including three catalytically active β5, β2, and β1 subunits as illustrated in Figures S4 and S5. In fact, the backbone of these N-terminal nucleophile hydrolases, despite significant differences in the amino acid sequence of their peptide chains (26 and 32% β5 homology with β2 and β1, respectively, and 35% between β2 and β1 subunit), align perfectly when structurally superimposed. In all catalytic subunits, two amino acids recognized as catalytic residues, i.e., Thr1 and Lys33, are preserved in the active sites together with the other 34 amino acids throughout the chain structure. Nonetheless, certain locations, especially near the active site, exhibit notable variations in the character of amino acid side chains at the same time scarcely affecting the protein folding. Importantly, some of these variations include significant changes in the local electrostatic environment. Thus, for instance, important reshuffling of the charge distribution is observed in the S1 pocket in position 45, where neutral methionine of β5 is exchanged by neutral glycine in β2 or positively charged arginine in β1. Another significant difference is found in position 53, where a neutral serine and glutamine of β5 and β1 correspond to negatively charged aspartic acid in β2. Importantly, the outcome of this redistribution directly affects the characters of the S1 pockets and, in consequence, the specificity of the binding channels. Despite variations in charge distribution, the collective electrostatic potential within the active site remains predominantly negative across three isolated catalytic subunits, ranging from −194.5 to −534.8 kJ·mol^–1^·e^–1^ (associated with β5 and β2, respectively). Nonetheless, each exhibits a negative Coulombic environment. Yet, because the reaction does not take place in the active site of the isolated proteins, the influence of the rest of the environment must be considered.

The vicinity of the three active sites is highly heterogeneous due to their location in the large 20S proteasome. Therefore, each of the active sites is surrounded by six or seven different proteins within the chosen cutoff distance, as illustrated in Figure 1E. All active sites face the interior of the cylinder and residue in a chamber formed by the centers of the abutting β-rings. Thus, the substrate binding cavity in the caspase-like enzyme is structurally shaped partially by β1 and the β2 subunit, in the trypsin-like protein by β2 and the β3 subunit, and in chymotrypsin-like by β5 and the β6. Therefore, it is possible that although not directly involved in the chemical transformation, proteins that coform substrate-binding cavities may participate in catalysis due to nonbonding interactions that could somehow affect the chemical process. Moreover, within a 20 Å radius from the active site, a value slightly higher than that usually selected for the nonbonding interaction cutoff (14–16 Å) used in the computational simulations of large systems, other subunits are present that can also contribute to the catalysis. Thus, the α5, β4, β6, β3′, and β4’ subunits are found in the vicinity of the β5 active site. In the case of β2, the α2, β3, β1, β5′, β6’, and β7’ are located, while the α1, β2, β7, β6′, β7’, and β1’ surround the active site of the β1 subunit. These different environments are responsible for the large deviation of electrostatic potential experienced by substrate (electrophile, C1 carbon atom) in the active site, i.e., + 188.8,–761.7, and +95.4 kJ·mol^–1^·e^–1^ in β1, β2, and β5, respectively. As observed, these values are significantly different to those computed for the three isolated catalytic subunits mentioned above. The contribution to the overall value of *V*_elec_ generated in each active site by a specific subunit is shown in Table S1 and Figure S6. Considering the spread of the calculated electrostatic potential values in each active site and assuming that they should correlate with the reaction mechanism and heights of the free energy barriers as demonstrated by previous studies^25,53^ the chemical process was subsequently explored and compared between all active sites.

### Mechanism of the Chemical Reaction

**Figure 2:**
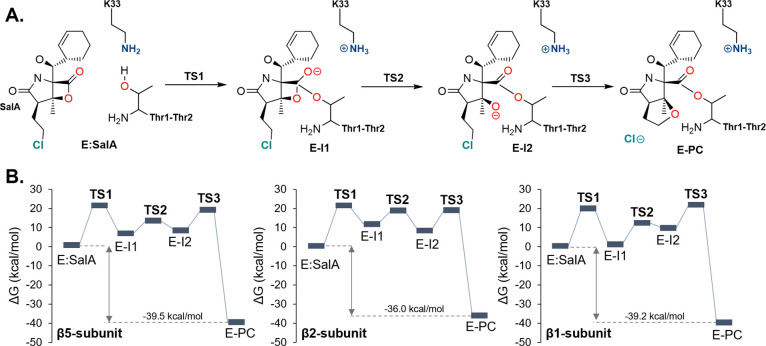
(A) Reaction mechanism explored in all catalytically active subunits. (B) Gibbs free energy profiles computed at M06-2X:AM1/MM level of theory for the 20S Proteasome inhibition with SalA in β5, β2, and β1 subunits. The energy profile for β5 was reproduced from ref (25). Copyright 2024 Americal Chemistry Society.

The mechanism of irreversible substrate-enzyme covalent adduct formation was explored in all three catalytically active subunits by using QM/MM MD methods. The definition of the QM region as well as details of the system setup are described in the Computational Methods Section. The same sequence of chemical transformations was assumed as originally proposed in our previous computational work in which the Lys33-assisted mechanism in the active site of the β5 subunit was found to be the most energetically favorable reaction path.^25^ Considering the results of crystallographic and kinetic studies,^52,53^ it has been proposed that the formation of the covalent ester bond between Thr1 and the β-lactone ring of SalA precedes the departure of the chlorine atom and the cyclic ether product formation. Therefore, the reaction mechanism was explored by computing two free energy landscapes (FELs). In the first one, the transfer of the proton (H^γ^) from Thr1 to nitrogen (N^ζ^) of Lys33 and the nucleophilic attack of O^γThr1^ on the carbonyl carbon (C1^SalA^) of β-lactone together with β-lactone ring opening were controlled. The ion pair intermediate (E-I2) generated during these transformations was subsequently used as the initial structure for the generation of the second FEL, where the formation of the O2^SalA^–C3^SalA^, bond and the breaking of the C3^SalA^–Cl^SalA^ leading to the formation of the THF ring and departure of the chloride atom were supervised. As shown in Figures S7–S9, the shapes of FELs computed for the chemical process occurring in all three β1, β2, and β5 subunits confirm that the reaction proceeds according to the same mechanism in all active sites. This mechanism (shown in detail in Figures 1C and 2), which involves three steps as confirmed by optimization and characterization of TS structures at the M06-2X^60^/AMBER^61^ level of theory, is characterized by very similar values of the computed rate-determining free energy of activation (Δ*G*^‡^), i.e., 20.4, 20.5, and 22.8 kcal/mol in β5, β2, and β1 subunits, respectively, but corresponding to different steps: the first step (the Thr1 nucleophilic attack) in β5 and β2 subunits, and the third step (the THF ring formation) in the β1 subunit. All computed free energy barriers agree well with rate constants (*k*_cat_) of 0.01, 0.003, and 0.02 s^–1^ measured experimentally for the studied process at 37 °C for β5, β2, and β1 subunits, respectively.^52^ The free energy profiles indicate the existence of two metastable intermediates (E-I1 and E-I2) formed during the reaction, which are completed by the formation of a very stable irreversible covalent complex (E-PC). In fact, a very similar energy stabilization of E-PC was found in all studied subunits.

The change of the r.d.s. in β1, by comparison with the free energy pathway for the same mechanism computed in β5 and β2, may be due to two reasons. The free energy barrier for the third step in β5 is lower than in β1or β2, probably because of a more advanced breaking stage of the C–Cl bond in the E-I2 structure, revealed by a more elongated distance (1.93 Å) in comparison to those in β2 and β1 (1.83 or 1.84 Å, respectively). On the other hand, even though C–Cl bonds are almost equal in the case of β2 and β1, the barrier for the latter is still higher by 1.7 kcal/mol. This energy shift could be due to the poor contacts established between the leaving group and the water molecules in the active site of β1 in the E-I2 (only two waters were found in the hydration shell) compared to four in the β2. Thus, this additional energy portion can account for the rearrangement of neighboring waters required to form proper ion hydration when chloride departs. For details, see Figure S10 representing the first hydration shell of the chloride in the E-I2 and E-PC.

**Figure 3:**
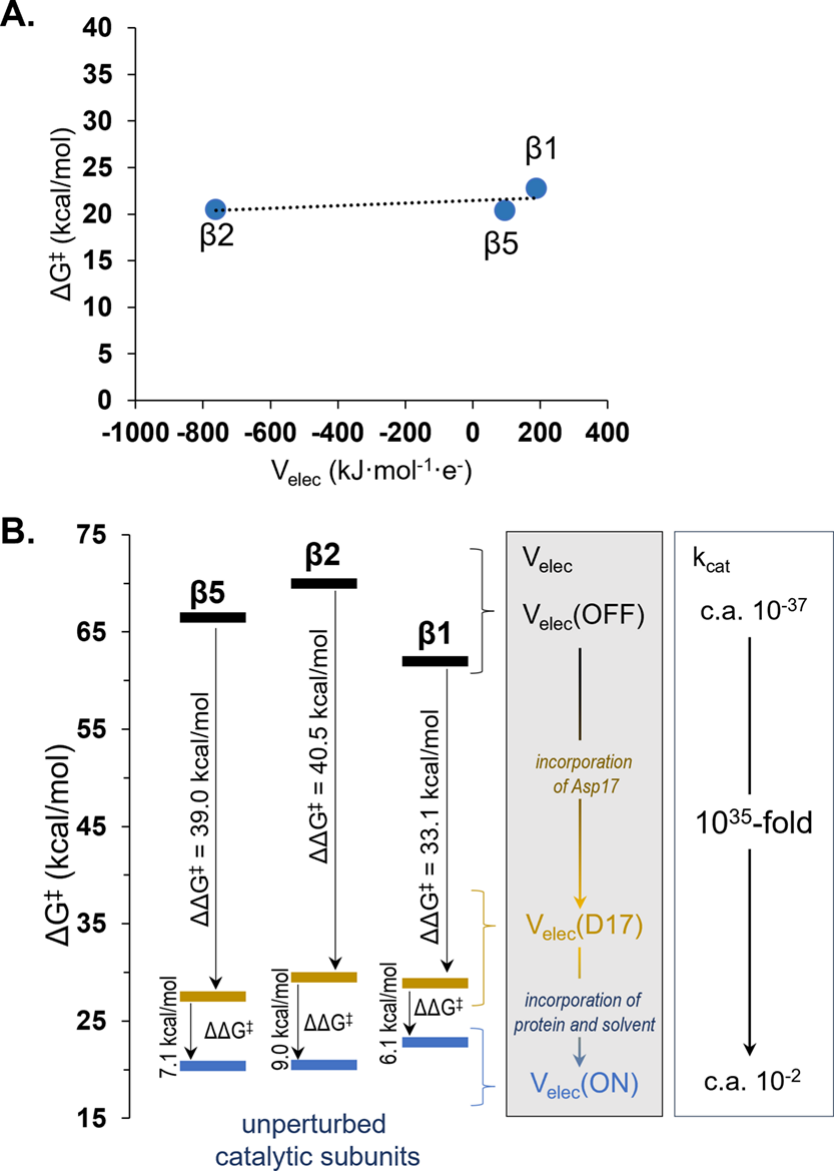
(A) Free energy barriers computed for rate-determining steps (rds) as a function of electrostatic potential generated in the active site of each catalytically active subunit. (B) Effect of electrostatic field atrophy and Asp17 on free energy barriers computed at the M06-2X:AM1/MM level for overall chemical transformations of SalA occurring in the active site of β5, β2, and β1 of 20S proteasome.

Unexpectedly, despite the electrostatic heterogeneity of the active site of each subunit, the catalytic efficiency was unperturbed, therefore resulting in the absence of any type of correlation between the calculated free energy barriers for r.d.s. and the values of the *V*_elec_ generated by the protein chains in the active site, as illustrated in Figure 3A. This result thus could suggest that the free energy barriers of the reaction taking place in 20S proteasome are completely independent of the magnitude of the external electrostatic field. This is a rather unexpected conclusion that utterly contradicted our initial hypothesis, leaving us with a difficult puzzle and, above all, the question of whether the electrostatic field indeed matters in catalysis.

### Role of the Electrostatic Field

#### Electrostatic Field Atrophy, *V*_elec_(OFF)

To revise if the Coulombic environment provided by the protein is meaningless for the enzymatic efficiency, the exploration of the FELs for all chemical events has been repeated in all three active sites, but this time excluding the point charges assigned in the MM force field to the atoms of amino acid residues, water, and counterions, similarly to the studies by Stare and co-workers on monoamine oxidase where the calculations were repeated in the gas phase.^29^ In our model, the only remaining charged residues were those directly involved in the chemical reaction, i.e., Lys33 and Thr1, and naturally the substrate, i.e. groups located in the QM region. However, the σ and ε parameters used to compute nonbonding Lennard-Jones potentials remained unchanged, ensuring that the conditions for the tested reaction were not vacuum. FELs computed in these conditions were used to determine the influence of the introduced changes on the reaction mechanism and free energy barriers (see Figures S7–S9). As proved, the atrophy of the electrostatic field in the active site resulted in the same reaction mechanism, but on the contrary, as shown in Figure 3B, drastic changes in the free energy profile were observed. In all subunits (see Table 1), the overall free energy barriers increased dramatically up to 66.5, 70.0, and 62.0 kcal/mol in β5, β2, and β1, respectively, which are now all related to the last, cyclization step. This incredible change in the activation barriers means nothing less than the enzymatic power was compromised and reduced by up to 10^35^ folds. Moreover, due to modification applied to the reaction environment, the chemical process in each active site evolved from highly exergonic to highly endergonic, directly indicating that *V*_elec_ must also play a meaningful role in the stabilization of the final product complex (E-PC). Further, analysis of the free energy profile for three consecutive reaction steps also revealed weaker stabilization of two intermediates E-I1 and E-I2, which directly contributes to the increase of the final energy barrier. Interestingly, in the case of the β5 subunit, the E-I1 structure completely lost its stability and was not observed anymore as a minimum on the surface. This was confirmed by IRC calculations, where the minimum energy path traced down from optimized and characterized TS2 in this specific variant of β5 subunit directly connected the structure of E-I2 with E:SalA.

**Table 1:** Relative Gibbs Free Energy for the Chemical Reaction of SalA with the Active Site of β5, β2, and β1 of 20S Proteasome Computed at the M06-2X:AM1/MM Level with Different Conditions of Electrostatic Potential (*V*_elec_) Generated in the Active Site^a^^,^^b^

	β5-subunit			β2-subunit			β1-subunit		
	*V*_elec_(ON)^c^	*V*_elec_(OFF)^d^	*V*_elec_(D17)^e^	*V*_elec_(ON)^c^	*V*_elec_(OFF)^d^	*V*_elec_(D17)^e^	*V*_elec_(ON)^c^	*V*_elec_(OFF)^d^	*V*_elec_(D17)^e^
E:SalA	0.0^g^	0.0	0.0	0.0	0.0	0.0	0.0	0.0	0.0
TS1	20.4^g^	32.4	18.5	20.5	34.2	19.6	19.4	31.5	17.3
E-I1	6.6^g^	32.0	8.5	11.8	33.8	8.0	1.2	30.5	6.3
TS2	13.8^g^	42.0	14.6	18.8	45.8	16.4	12.7	40.8	15.1
E-I2	9.1^g^	39.1	11.5	8.7	42.6	13.0	9.8	38.6	11.9
TS3	18.8^g^	66.5	27.5	20.0	70.0	29.5	22.8	62.0	28.9
E-PC	–39.5^g^	55.5	9.9	–36.0	47.0	2.5	–39.2	35.6	–9.1

	experimental data		
*k*_inact_ (Δ*G*^‡^)	0.01 s^–1^^f^ (20.6 kcal/mol)	0.003 s^–1^^f^ (21.6 kcal/mol)	0.02 s^–1^^f^ (20.5 kcal/mol)

a The activity of enzyme subunits computed in different electrostatic conditions, i.e., in.

b Values of energies are given in kcal/mol.

c An unperturbed *V*_elec_ generated in the active site by amino acid residues within the range of cutoff distance.

d Turned-off *V*_elec_, despite two catalytically involved Lys33 and Thr1 residues.

e With *V*_elec_ generated by Asp17.

f Rates of inactivation measured experimentally at *T* = 37 °C obtained from ref (52) with corresponding free energy barriers deduced based on transition state theory.

g Those values are taken from ref (25).

#### Electrostatic Field of Individual Residues

**Figure 4:**
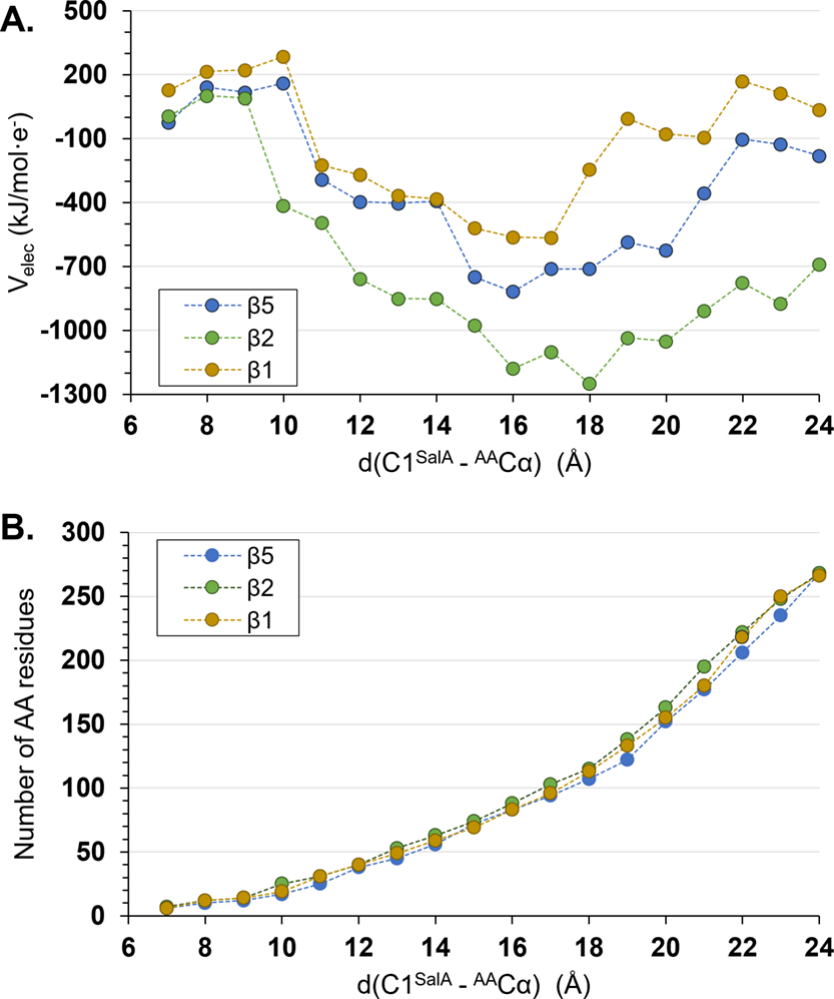
(A) Electrostatic potential, *V*_elec_ generated by amino acid residues within a selected radius from carbon C1 of SalA bound in the active sites of β1, β2, and β5 subunits 20S proteasome. (B) Number of AA residues within the selected distance that contribute to the computed *V*_elec_ values.

The results obtained under electrostatic field atrophy conditions indicated that there must exist a more specific dependency between the speed of the reaction and the charge distribution in the protein. As it was impossible to deduce any correlation between the overall V_elec_ generated in the active site and free energy profiles, we decided to compute the *V*_elec_ generated in the active site decomposed by residue. The values of computed *V*_elec_ can be found in Tables S2–S4. Subsequently, we have ordered the residues according to the distance from the active site, using as the reference position of the carbon C1 (electrophilic center) of SalA and the carbon Cα of the backbone of each residue. The change of *V*_elec_ in the protein was monitored between 7 and 24 Å with an interval of 1.0 Å. As shown in Figure 4A, *V*_elec_ generated by each residue does not oscillate randomly when increasing the distance to the electrophile of the substrate located in the active site. In addition, a similar pattern of both: the evolution of *V*_elec_ and the increase of the number of amino acid residues (see Figure 4B) while moving away from the active site of every catalytic subunit, is observed. Thus, it was found that until ∼9 to 10 Å in all subunits, the *V*_elec_ is positive and afterward drops rapidly to highly negative values. Negative *V*_elec_ is subsequently neutralized from 17 to 18 Å, where it starts to increase again. This important shift in the magnitude and the sign of *V*_elec_ prompts questions about the appropriate magnitude for the cutoff for nonbonding interactions to be used in the calculations of enzymatic systems.

**Figure 5:**
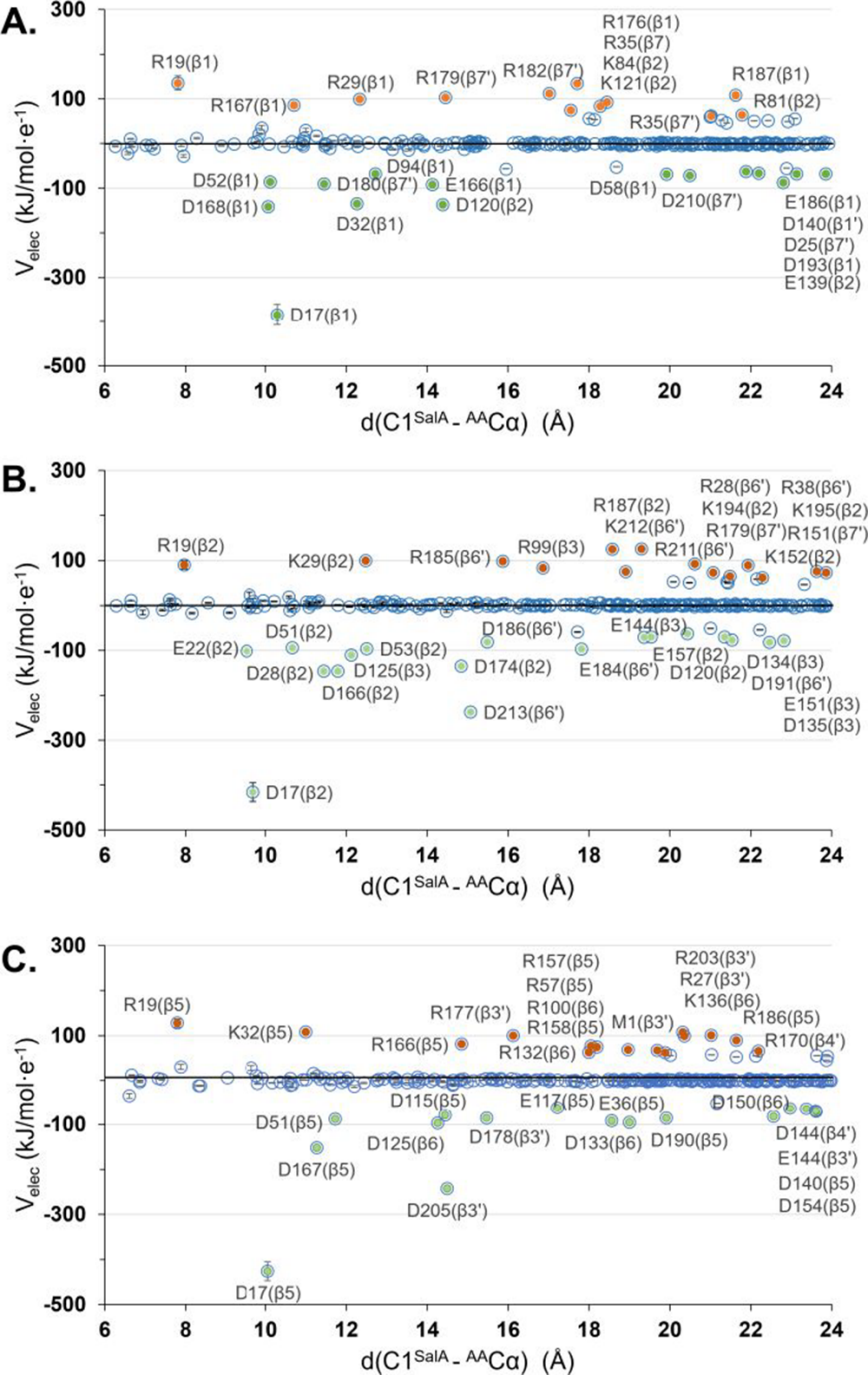
Individual contribution of amino acid residues located within the selected radius from carbon C1 of SalA, to electrostatic potential, *V*_elec_, generated in the active sites of β1 (panel A), β2 (panel B), and β5 (panel C), subunits of 20S proteasome. Residues contributing to positive *V*_elec_ are highlighted in orange, and those contributing to negative *V*_elec_ are shown in green. Blue points represent residues within the selected distance with no significant electrostatic contribution. Only residues that contribute more than 60 kJ/mol·e^–1^ in absolute value are labeled.

We decided to examine this problem by considering the same protocol as originally used in our laboratory to redesign the promiscuous esterase from *Bacillus subtilis* Bs2 to enhance its secondary amidase activity.^26^ The method basically consists of stabilizing one of the key states appearing in the reaction path by mutations that generate electrostatic potentials that stabilize the increase or decrease in the electron density of the reacting systems. In the present system, we focused on finding those residues that stabilize the positive charge accumulated on the nucleophile activator, Lys33. Therefore, we analyze the first shell around the active site that generates negative *V*_elec_, i.e., between 9 and 10 Å in β2 and 10 and 11 Å in β5 and β1. (Figure 4A) The residues located within this range were isolated, and their contribution to the stabilization of positive charge accumulated on Lys33 in E-I1 was estimated. As shown in Figure 5A–C, the dominant contribution to the overall negative *V*_elec_, within the chosen distance range, is played by Asp17 in all cases. This residue is preserved among 34 identical residues present in all of the catalytic subunits. Its contribution to the negative *V*_elec_ ranges between 94 and 75% depending on the subunit. Moreover, geometrical inspection revealed that this amino acid is in very close hydrogen bond contact with Lys33, the residue responsible for the activation of the nucleophile. In fact, Asp17 residue was already proposed by us^25^ as well as by recent computational studies of Huang and co-workers^59^ as a key residue for the catalytic process. Taking all these results into account, we decided to explore FELs once again, this time deleting all point charges of the protein residues but restoring partial charges on Asp17, as defined in the MM force field, in all catalytic subunits. The results of these calculations are provided in Figures S7–S9. Unexpectedly, as shown in Table 1 and in Figure 3B, it was found that the *V*_elec_ generated by Asp17 was not only contributing to the free energy barrier reduction but restoring the charge of Asp17 was enough to regain the catalytic power for the two first chemical transformations that occur in every considered β-subunit, i.e., the nucleophilic attack and the β-lactone ring opening. This unexpected one-residue effect may suggest that Thr1, Lys33, and Asp17 form the catalytic triad that is placed in the perfect position to catalyze the formation of the enzyme-SalA complex. Asp17’s role is to stabilize the positive charge accumulated on Lys33 during the first step, thus improving stabilization of the E-I1 that according to Hammond’s postulate^62^ can be responsible for reducing the barrier of the first TS. It must be pointed out that the inclusion of atomic charges located on Asp17 resulted in all cases in slight reduction of Δ*G*^‡^ of the first step compared to barriers observed in electrostatically unperturbated catalytic subunits. This modest reduction of the free energy barrier of the first step is probably due to the absence of the positive *V*_elec_ generated by Arg19 (residue preserved in all subunits) that, as shown in Figure 5, would compensate, to a small extent, for the overall influence of Asp on the catalysis. However, the presence of Arg19 is not enough to disturb the enormous impact of Asp17 on the catalysis of the tested reaction.

This outcome supports our original strategy used to redesign Bs2, by comparison with a nonhomologous amidase *Candida antarctica* lipase B (CALB), where the stabilization of the positive charge accumulated in the catalytic His224 (residue with acid/base role in a catalytic triad) during the first step reaction resulted in a meaningful barrier reduction of the nucleophilic attack.^24^ The same situation takes place here, proving that the stabilization of E-I1 is not only crucial for the first step but also directly affects C–O bond breaking, which mimics peptide bond scission of the primary process. Nevertheless, although the enzymatic efficiency for the two steps of the reaction was almost fully recovered by the inclusion of Asp17, the last step was not.

Intramolecular cyclization accompanied by the release of chlorine anion (Cl^–^) is the artificial process that takes place inside the active site that was originally not designed by Nature for its support. Therefore, we envisioned that the destabilization of the final product complex (E-PC) still observed in the enzyme variant with charged Asp17, must be mostly due to a lack of favoring interaction between solvent and chlorine anion. Thus, to prove it, the charges on water molecules were switched back on, and the FELs for the last step were recalculated for each active site (see Figures S11–S13). Indeed, the contribution of the solvent was confirmed to play a significant role in increasing the stabilization of the E-PC in all β-subunits, as shown in Table 2.

**Table 2:** Relative to E-I2 Free Energy Barriers and Energy of Stabilization of E-PC in Different Electrostatic Conditions for Active Site Inactivation with SalA in β5, β2, and β1 Subunits^a^^,^^b^

	*V*_elec_(ON)^c^	*V*_elec_(OFF)^d^	*V*_elec_(D17)^e^	*V*_elec_(D17&WAT)^f^
β5-subunit				
TS3	8.9	27.4	16.0	10.1
E-PC	–48.6	16.4	–1.6	–35.1
β2-subunit				
TS3	11.3	27.4	16.5	13.1
E-PC	–44.7	4.4	–10.5	–30.6
β1-subunit				
TS3	13.0	23.4	17.0	8.1
E-PC	–49.0	–3.0	–21.0	–73.5

a The activity of enzyme subunits computed in different electrostatic conditions, i.e., in.

b Values of energy are given in kcal/mol.

c An unperturbed *V*_elec_ generated in the active site by amino acid residues within the range of cutoff distance.

d Turned-off *V*_elec_, despite two catalytically involved Lys33 and Thr1 residues.

e With *V*_elec_ generated by Asp17.

f With *V*_elec_ generated by Asp17 and water molecules.

This strong effect of the solvent can be justified by the fact that in all β-subunits, the leaving chloride departs directly to the water-rich channel, where it is immediately hydrated. In all cases, between five and six dynamic water molecules are found in the proximity of the Cl anion in the product complex (E-PC), in agreement with previous work on the molecular structure of chloride in aqueous solution by Hunt and co-workers.^63^ Accordingly, 4 strong H-bond interactions are formed in its first hydration shell (as shown in Figure S10). Our results confirm that when chloride is still attached to the inhibitor, its interactions with water molecules are weaker, with no direct H-bond contacts.

Nevertheless, the original thermodynamic stabilization of the E-PC was never reached, as shown in the case of β5 and β2-subunits when charges were switched back on. On the contrary, overstabilization of E-PC was observed in β1. Both results suggest that there must exist an additional effect originating in the specific protein environment that contributes to the final value of the reaction-free energy observed in unperturbed enzymes. In order to confirm this hypothesis, we focused on the β1 case, and consequently, *V*_elec_ generated by the protein on the chlorine atom, decomposed by residue, was recalculated in the structure of E-PC. The negatively charged Asp167 was found as the only amino acid residue within 12 Å from the Cl^–^ ion (see Table S5) that could be responsible for the destabilization of the E-PC structure. Therefore, a new electrostatic variant of β1 was created with force field point charges assigned to Asp17, Asp167, and water molecules. A new FEL was computed for the third step of the process within this model, D17&WAT. As predicted, the inclusion of Asp167 resulted in the destabilization of E-PC reflected by an energy shift from −73.5 kcal/mol in *V*_elec_(D17&WAT) variant to −46.3 kcal/mol, a value that is very close to the final −49 kcal/mol, observed for E-PC in the unperturbed enzyme. Undeniably, our initial guess based exclusively on values of *V*_elec_ generated individually by each residue appeared to be very effective in forecasting the impact of a specific amino acid on the thermodynamics of the process. Therefore, this suggests that the analysis of local *V*_elec_ can be useful in recognizing the key elements of complex protein structures specifically vital for catalysis.

#### Electrostatic Field Variations

**Figure 6:**
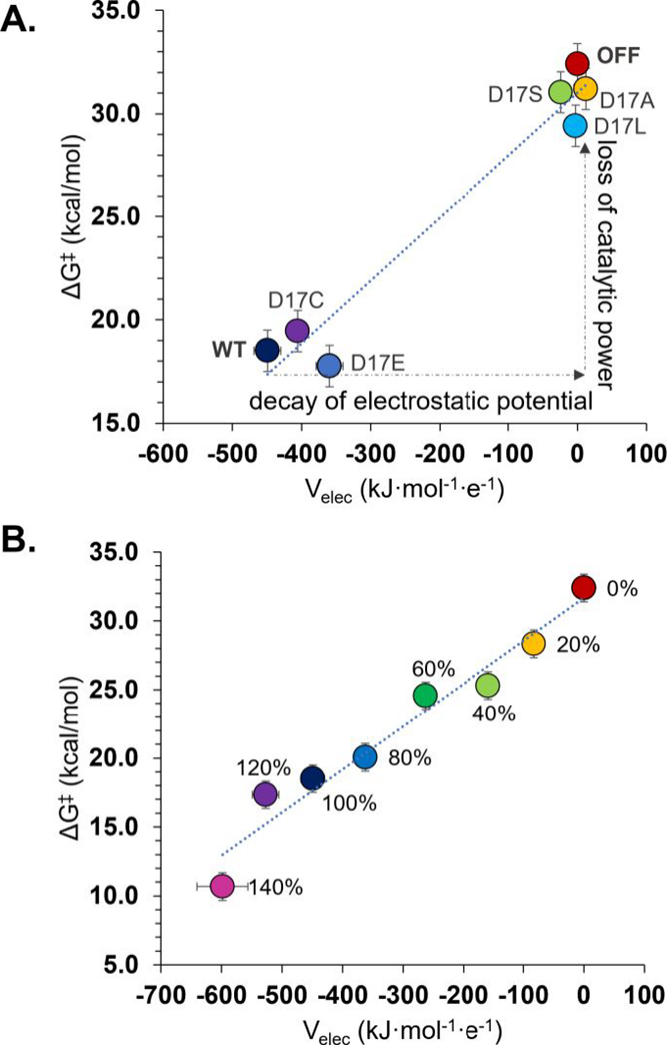
(A) Free Gibbs energy barriers as a function of the electrostatic potential, *V*_elec_ generated on nucleophile activator (N^ζ^ of Lys33) in the active site of β5 subunit by its different D17X variants, where X = Ala, Ser, Leu, Cys, and Glu. Wild type (WT) corresponds to the electrostatically unperturbed Asp17 variant in the β5-subunit, while the point labeled as OFF corresponds to the barrier obtained when annihilating all the charges of the environment. (B) Free Gibbs energy barriers as a function of the electrostatic potential, *V*_elec_ generated in the active site of the β5 subunit by changing the contribution of point charges of Asp17.

The obtained results clearly indicate that changes in the electrostatic characteristics of the environment (within 12 Å) indeed have a dramatic impact on the stabilization of the TSs, intermediates, and products and consequently on the kinetics and thermodynamics of the enzymatic process. To shed more light on the role of Asp17 on the catalysis, we decided to perturb the electrostatic potential generated by the residue in this position in the active site of the β5 subunit using two different additional approaches. In the first one, several mutations were proposed at position 17. Therefore, Asp17 originally present in WT was substituted by several natural neutral amino acids, such as Ser, Ala, and Leu, and negatively charged deprotonated Cys and Glu. After mutation, FELs for the first step were recomputed (see Figure S14), providing new values of Δ*G*^‡^ and demonstrating how the changes in Δ*G*^‡^ were induced by mutagenesis. It is worth mentioning that for this in silico experiment, we completely neglected the possible influence of mutations on the conformational protein structure change. As observed in Figure 6A, the values of *V*_elec_ drastically decrease upon the substitution of negatively charged Asp17 by neutral amino acids increasing meaningfully the values of Δ*G*^‡^. (see Figure 6A) The value of computed *V*_elec_ remains within the range of that found in the active site of WT only when Asp is mutated to negatively charged Cys or Glu. Computed barriers for WT, D17C, and D17E variants are very similar (considering the 1 kcal/mol error assigned to the employed computational approach). The correlation between the *V*_elec_ and the free energy barriers found in this approach can be used to rationalize the effects measured on rate when site-directed mutagenesis experiments are performed on the vicinity of the substrate.^28,64^

Unfortunately, as can be seen in the present in silico experiments, the use of natural amino acids limits the repositories of chemical groups and consequently restrains the range of *V*_elec_ that can be applied in catalysis, thus not allowing for the exploration of a wide range of possibilities. In the case of the 20S proteasome, mutation of Asp can result only in an inactivated or similarly fast variant of the enzyme. Therefore, in the second approach, we tried to fill this gap by an analysis that could not be met by the introduction of natural amino acids. Here *V*_elec_ generated by Asp17 was gradually switched off by introducing a scaling factor λ (defined in the range from 0 to 1.4 with an interval of 0.2) on partial charges provided in the force field to this specific residue. As shown in Figure 6B, a very good linear correlation between *V*_elec_ and the free energy barriers (determined based on FEL presented in Figure S15) was obtained, confirming that there is a direct relationship between enzymatic catalysis and local electrostatic provided by protein. It was also demonstrated that an unnatural increase of charge on Asp17 by 20 or 40% results in a meaningful catalytic improvement, which is reflected by the reduction of the free energy barrier for the first step by 6.9 kcal/mol with respect to the unperturbed β5 variant.

### Spatiotemporal Theory

**Figure 7:**
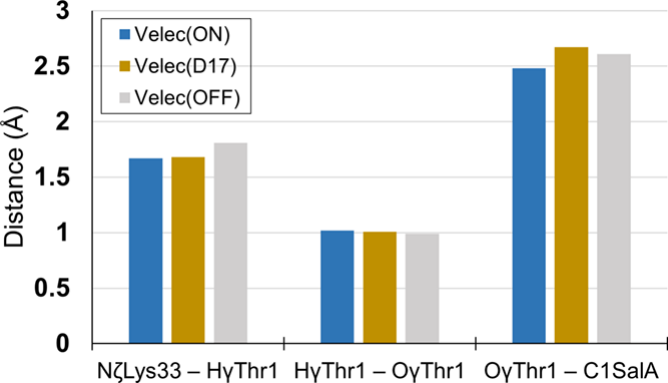
Key distances in E:SalA complex in alternative electrostatic variants of β5-subunit.

Finally, the simpler concept, i.e., Spatiotemporal theory,^65^ was also considered in this work. This alternative proposal, similar to the compression effect hypothesis,^11^ suggests that to enhance chemical reaction, two functionalities must be held rigidly at contact distances less than ca. 3 Å. Structural analysis of our results (as shown in Figure 7) indicates that in the case of 20S proteasome active sites, the close contact between the hydrogen donor and acceptor or electrophile and nucleophile centers is not sufficient to achieve the catalytic power. Although all reacting groups are within close contact, still large differences in the free energy barriers can be found. Nevertheless, it is intuitively indisputable that the proximity of the groups involved in a chemical reaction is the basic requirement for the reaction to occur. In our understanding, both spatiotemporal and electrostatic preorganization hypotheses complement rather than compete with each other. According to spatiotemporal theory, in enzymatic catalysis, the protein scaffold must first ensure the appropriate position of the binding compounds in the active site (Michaelis complex formation) that is further complemented by the electrostatically appropriate oriented environment to enable fast chemical transformation (increase of rate constant, *k*_cat_).

## Conclusions

The results of this work confirm that the enzyme active site provides a preorganized local polar environment that stabilizes the TS, regardless of the polarizability of the overall scaffold of the protein that anchors the key catalytic residues and is not necessarily limited to the active site residues in direct contact with the substrate. In the case of the 20S Proteasome, it has been demonstrated that three catalytic subunits, significantly different from the point of view of sequence homology and with active sites located in the electrostatically miscellaneous environment, can speed up the process of transformation of SalA in a very similar way, in agreement with experimental data. The similar catalytic efficiency is due to residues located within 12 Å from the reactive site of the substrate and, in particular, to the presence of only three key residues, i.e., Thr1, Lys33, and Asp17. It is the presence of Asp17 that, besides the two amino acids directly involved in the SalA-inactivation reaction catalytic residues, is required to improve the efficiency of the process by 10^35^ folds, by comparison to the counterpart reaction in an alchemical enzyme with neglected charges. Mutation of this residue results in the inactivation of the enzyme, except in the case of mutation to negatively charged Cys and Glu.

The obtained results are in agreement with conclusions previously obtained in studies on the monoamine oxidase system with the DFT cluster approach,^29^ and can suggest that any mutation above this distance, as many times generated during directed evolution experiments, plays a conformational or stabilizing role on the protein structure rather than influencing the rate (*k*_cat_) of the chemical process itself. Nevertheless, as mentioned, the 3D structure and flexibility of the full biocatalytic machinery are required to keep the electrostatically relevant residues in proper spatial positions relative to the substrate. Distant mutations may ultimately meaningfully affect the conformation of the protein, affecting not only the spatial distribution of the key identified residues relevant for the chemical steps but also the substrate binding process, therefore influencing the overall Michaelis–Menten kinetic. From the computational point of view, we stress the relevance of the selected cutoff criteria.

Even being amazing catalysts, based on the results of this study, it can be concluded that enzymes still do not necessarily exhaust all the possibilities in accelerating chemical reactions, due to the limited types of chemical groups that can be provided by natural amino acids used for their synthesis. As demonstrated by in silico modifications, chemical groups provided with the available side chains of amino acid residues cover just a small area of manipulation that could be achieved by the introduction of artificial groups. Such unnatural modifications could allow for achieving much better performance without necessarily losing key structural characteristics of the protein. In fact, this area is being recently explored by developing new techniques used to engineer proteins with noncanonical amino acids.^66^ However, a note of caution must be introduced since, as demonstrated in the present study, the structure of the full protein machinery is crucial, and introducing new unnatural residues could compromise it, with the consequent effects on the reaction to be catalyzed.

Finally, the method proposed in this work, consisting of theoretical isolation of the value of the electrostatic potential generated at key points of the active site by individual amino acid residues of the complex polypeptide matrix, makes it possible to reveal key residues for catalysis and therefore can prove to be vital for enzyme-based catalysts design in a rational design approach.

## Computational Methods

### System Setup

The 20S proteasome model was prepared based on the model used in our previous studies.^25,57,67,68^ The original crystal structure of the 20S proteasome was obtained from the Protein Data Bank (PDB ID: 5LF1).^47^ The dihydroeponemycin molecule bound originally in the active site of the β5 subunit of the structure was substituted by the covalently bound product of the inhibition by SalA. The orientation of the inhibitor in all three catalytically active sites was adapted from the crystal structure of 20S proteasome from *Saccharomyces cerevisiae* complexed with this molecule (PDB ID: 2FAK).^53^ Additionally, three chlorine atoms, not present in the crystal structure of the final product, were manually added to the system. Missing force field parameters for the inhibitor and its covalent adduct were adapted from the previous studies.^25^ The protonation state of the titratable residues at pH 7 was predicted as described in a previous work^57^ using the PropKa program ver. 3.1,^69,70^ and missing hydrogen atoms were added using the tLEAP module of the Amber Tools package.^71^ The neutralization of the system was achieved by adding 43 sodium counterions at the electrostatically most favorable positions. Finally, the full protein together with counterions was solvated in an orthorhombic box of TIP3P^72^ water molecules of 17.2 × 16.9 × 19.7 nm^3^, providing a model containing more than half a million of atoms.

### MM MD Simulations

After preliminary minimization using an AMBER force field with NAMD^73^ software, the system was heated to 310 K with a 0.001 K temperature increment. A 100 ps NPT equilibration was carried out with nonbiased MD simulations. Then, positions of all residues located beyond 40 Å from the inhibitor were frozen and 100 ps of the additional equilibration under NVT conditions were applied. First MD simulations were done in the product complex state, allowing the structure of the protein to adapt to the presence of the inhibitor. Because the chlorine atom is free in the final product, its position was fixed at 6 Å from the inhibitor to avoid its diffusion from the active site to the bulk solvent during the simulations. Finally, 5 ns of nonaccelerated unbiased NVT MD simulations were performed. The final structure was then modified to prepare the Michaelis complex structure. This new reactant complex served as a starting geometry for 50 ns of nonaccelerated NVT MD simulations. In order to model SalA in the β2 and β1 active sites, the reactant complex was modeled by overlapping the β5 reactant complex along with the conserved active site residues, Thr1-Thr2. Therefore, the simulation for these two active sites could be started from the Michaelis complex structure. The temperature during the simulations was controlled using a Langevin thermostat.^74^ In order to improve the time of simulations, cut-offs for nonbonding interactions were applied using a smooth switching function between 14.5 and 16 Å. The systems were considered equilibrated due to the RMSD and analysis of the evolution of key distances along the MD simulations. All data from MD simulations were provided in ref (25).

### QM/MM Calculations

Consequently, we have followed the same protocol as in the previous study.^25^ Thus, an equilibrated active conformer structure was selected to explore the inhibition mechanisms in each system (β2 and β1). In order to improve the time of the QM/MM calculations, the full protein was kept in the model, but the solvent was reduced to a sphere of 60 Å radius centered on the Thr1 residue of each corresponding subunit, thus decreasing the total number of atoms in the system by about 70%. A small portion of the system, i.e., the full inhibitor, Thr1, part of Thr2, and the side chain of Lys33, was described by the AM1 semiempirical Hamiltonian or the M06-2X DFT functional with the standard 6-31+G(d,p) basis set. The protein, counterions, and solvent water molecules were described by AMBER and TIP3P force fields, as implemented in the fDynamo library.^75,76^ Two link atoms were inserted on the QM/MM boundary, intersecting covalent bonds. These were placed between the Cα–Cβ bond of Lys33 and the C–Cα bond of Thr2. For details, see Figures S16 and S17.

The QM region consists of 85 atoms in the three systems. The positions of the atoms of residues beyond 20 Å from the inhibitor were frozen, and the same cut-offs as in MD simulations were applied for the nonbonding interactions. Potential energy surfaces were obtained by grid scanning of appropriate distinguished reaction coordinates in each of the chemical steps. Based on these surfaces, structures of stationary points of the reactant complex (E:SalA), intermediates (E-I), product complex (E-PC), and TS structures were optimized using a micro–macro iteration method^77^ at AM1/MM and M06-2X/MM levels and characterized by computing the matrix of second energy derivatives. IRC paths were traced down from the M06-2X/MM TSs to further confirm that they connected the expected minima. Key distances of stationary structures localized in this study are provided in Tables S6–S11.

### Free Energy Surface (FES)

Reaction-free energy profiles were obtained based on FESs explored in terms of potentials of mean force (PMF). The PMF calculation requires a series of molecular dynamics simulations using a limited number of distinguished reaction coordinate variables that are constrained during the simulation. All PMFs were initially calculated at the AM1/MM level using the weighted histogram analysis method (WHAM)^78^ combined with the umbrella sampling (US)^79^ approach as implemented in fDYNAMO. The value of the force constant used for the harmonic umbrella sampling to generate the PMFs was 2500 kJ mol^–1^ Å^–2^, and the simulation windows consisted of 5 ps of equilibration and 20 ps of production, with a time step of 1 fs. WHAM calculations converged with a 1 × 10^–3^ density tolerance. Altogether, more than 40,000 US windows were used for the results presented in this work, as well as the same number of the corresponding single-point calculations at a high M06-2X/MM level of theory.

### Spline Corrections

To reduce the errors associated with the quantum level of theory employed in our simulations, a new energy function defined in terms of interpolated corrections was used.^80−82^
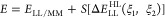
1where S denotes a two-dimensional spline function, and its argument is a correction term evaluated from the single-point energy difference between a high-level (HL) and a low-level (LL) calculation of the QM subsystem. The AM1 semiempirical Hamiltonian was used as the LL method, while the hybrid M06-2X functional with the standard 6-31+G(d,p) basis set was selected for the HL calculation. These calculations were carried out using the Gaussian09 program^83^ in combination with fDynamo.

### Electrostatic Potential

Electrostatic potential was calculated using the classical expression:
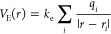
2where *r* is a point at which the potential is evaluated, *r_i_* is a point at which there is a nonzero charge, and *q_i_* is the charge at the point *r_i_*
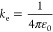
3where ε_0_ is the vacuum permittivity.

### System Modifications

To understand if the electrostatics of the protein have any significance for the chemical process taking place in the active site, we modified the part of the AMBER force field parameter files that assign atomic charges to the atoms of amino acid residues. So, the exploration of the FESs was repeated for all three subunits, simultaneously excluding atomic charges assigned to all atoms of protein residues, water molecules as well as counterions in the *V*_elec_(OFF) variant. The only charge carriers in this case were the substrate and two key residues for catalysis, i.e., Thr1 and Lys33, due to their presence in the QM region. The σ and ε parameters used to compute nonbonding Lennard-Jones potential remained unchanged. Depending on the course of the study, subsequently, the charges were switched on for Asp17, Asp167, or water molecules, as explained in the main text. For studies involving a mutation of Asp17, the replacement of residue to Cys, Ala, Leu, and Ser was done automatically using PyMOL ver.2 software.^84^ Finally, the decay of Asp17’s contribution to the electrostatic effect was controlled by applying the λ factor (from 0.2 to 1.4) that affected the final values of point charges assigned to the atoms forming the amino acid residue. Values of used point charges are listed in Table S12.

## Supporting Information Available

Schemes providing the structure of the active site, structural analysis of α and β subunits, and sequence alignment as well as interaction diagrams; figures presenting free energy surfaces computed for β1, β2, and β5-subunits in different electrostatic conditions; tables collecting point charges assigned to Asp17, the contribution of 20S proteasome subunits in *V*_elec_, the contribution of individual amino acid residues in *V*_elec_ generated on the electrophile in the E:SalA complex, *V*_elec_ generated per amino acid residue on Cl- leaving in E-PC complex, key distances for localized stationary points along the reaction progress; and cartesian coordinates for all optimized structures at M06-2X/MM level together with values of imaginary frequencies for TSs as well as results of IRC calculations provided in two additional separate files (PDF)
MEPs (XLSX)
Structure coordinates (XLSX)
