# Supplementary material for: Electrostatic Preorganization in Three Distinct Heterogeneous Proteasome β-Subunits
Source: ACS Catal. 2024 Oct 2;14(20):15237–49. doi: 10.1021/acscatal.4c04964 (PMC11494509; doi:10.1021/acscatal.4c04964)
Supplement: Supplementary file 1 — cs4c04964_si_001.pdf [file cs4c04964_si_001.pdf]

Supporting Information:

Electrostatic Preorganization in three distinct heterogeneous Proteasome β-Subunits.

Silvia Ferrer, Vicent Moliner, Katarzyna Świderek*

BioComp group, Institute of Advanced Materials (INAM), Universitat Jaume I, Avenida de Vicent Sos Baynat, s/n, 12071 Castellón, Spain.

*corresponding author: K.Ś. Email: swiderek@uji.es

Table of contents:

1. Structural analysis of α and β subunits. 3

1.1 Structural and sequence alignment 3

1.2 Contribution of 20S Proteasome subunits in V_{elec} generated in each active site 7

2. Mechanism of reaction in three catalytically active subunits of the 20S proteasome. 8

2.1 Free energy surfaces (FESs) computed for β1-subunit 8

2.2 Free energy surfaces (FESs) computed for β2-subunit 9

2.3 Free energy surfaces (FESs) computed for β5-subunit 10

3. The hydration shell of the chloride in the E-I2 and E-PC structures. 11

4. Contribution of individual amino acid residues in electrostatic potential (V_{elec}) generated on the electrophile in the E:SalA complex 12

4.1 Electrostatic potential (V_{elec}) in β5-subunit 12

4.2 Electrostatic potential (V_{elec}) in β2-subunit 15

4.3 Electrostatic potential (V_{elec}) in β1-subunit 18

5. Electrostatic potential (V_{elec}) generated per amino acid residue on Cl- leaving in the E-PC complex. 21

6. Stabilization of the final product of the reaction 24

6.1 Free energy surfaces (FESs) computed for β1-subunit 24

6.2 Free energy surfaces (FESs) computed for β2-subunit 24

6.3 Free energy surfaces (FESs) computed for β5-subunit 25

7. Electrostatic field variations 26

7.1 Free energy surfaces (FESs) computed for β5-subunit at D17AA variants 26

7.2. Free energy surfaces (FESs) computed for β5-subunit at D17X variants 27

8 Computational methods 28
8.1 Structure of the active site 28
8.2 Interactions diagrams of the active sites of each catalytic subunit 29
9 Key distances of optimized stationary points 30
9.1 Structures of optimized stationary points in V_{elec}(ON) conditions 30
9.2 Structures of optimized stationary points in V_{elec}(OFF) conditions 31
9.3 Structures of optimized stationary points in (V_{elec}(D17) conditions 31
10. System modifications 32
11 References 33

1. Structural analysis of α and β subunits.

1.1 Structural and sequence alignment

```
Beta5      TTTLAFKFRHGVIVAADSRATAGAYIASQTVKKVIEINPYLLGTMAGGAADCSFWERLLA      60
Beta2      TTIAGVVYKDGIVLGADTRATEGMVVADKNCSKIHFISPNIYCCGAGTAADTDMTTQLIS      60
Beta1      TTIMAVQFDGGVVLGADSRTTTGSYIANRVTDKLTPIHDRIFCCRSGSAADTQAVADAVT      60
           **  .. :  *:::.**:*:* *  :*.:  .*:  *   :    :* *** .     ::

Beta5      RQCRIYELRNKERISVAAASKLLANMVYQYKGMGLSMGTMICGWDK-RGPGLYYVDSEGN      119
Beta2      SNLELHSLSTGRLPRVVTANRMLKQMLFRYQGY-IGAALVLGGVDV-TGPHLYSIYPHGS      118
Beta1      YQLGFHSIELNEPPLVHTAASLFKEMCYRYRED-LMAGIIIAGWDPQEGGQVYSVPMGGM      119
            :  ::.:   .   * :*  :: :* ::*:   :  . :: * *   *  :* :   *

Beta5      RISGATFSVGSGSVYAYGVMDRGYSYDLEVEQAYDLARRAIYQATYRDAYSGGAVNLYHV      179
Beta2      TDKLPYVTMGSGSLAAMAVFEDKFRPDMEEEEAKNLVSEAIAAGIFNDLGSGSNIDLCVI      178
Beta1      MVRQSFAIGGSGSSYIYGYVDATYREGMTKEECLQFTANALALAMERDGSSGGVIRLAAI      179
                    ****    . .:  :  .:  *:. ::. .*:  .  .*  **. : *  :

Beta5      REDGWIRVSSDNVADLHEKYSGSTP------------------------------  204
Beta2      SKNKLDFLRPYTVPNKKGTRLGRYRCEKGTTAVLTEKITPLEIEVLEETVQTMDTS  234
Beta1      AESGVERQV-----------------------LLGDQIPKFAVATLPPA-------  205
            :.
```

Figure S1. Alignment of the amino acid sequences of catalytic β subunits of 20S Proteasome from *Homo Sapiens.* All conserved amino acid residues are highlighted by (*) star symbol. Polar amino acids are highlighted in green, non-polar in red, acidic in blue and basic in pink.

```
Beta1       ---------TTIMAVQFDGGVVLGADSRTTTGSYIANRVTDKLTPIHDRIFCCRSGSAAD      51
Beta2       ---------TTIAGVVYKDGIVLGADTRATEGMVVADKNCSKIHFISPNIYCCGAGTAAD      51
Beta3       -MSIMSYNGGAVMAMKGKNCVAIAADRRFGIQAQMVTTDFQKIFPMGDRLYIGLAGLATD      59
Beta4       --------MEYLIGIQGPDYVLVASDRVAASNIVQMKDDHDKMFKMSEKILLLCVGEAGD      52
Beta5       ---------TTTLAFKFRHGVIVAADSRATAGAYIASQTVKKVIEINPYLLGTMAGGAAD      51
Beta6       RFSPYVFNGGTILAIAGEDFAIVASDTRLSEGFSIHTRDSPKCYKLTDKTVIGCSGFHGD      60
Beta7       -TQNPMVTGTSVLGVKFEGGVVIAADMLGSYGSLARFRNISRIMRVNNSTMLGASGDYAD      59
                         ..       :.:*               :   :         *   *

Beta1       TQAVADAVTYQLGFHSIE-LNEPPLVHTAASLFKEMC--YR-YRE-D-LMAGIIIAGWDP      105
Beta2       TDMTTQLISSNLELHSLS-TGRLPRVVTANRMLKQML--FR-YQG-Y-IGAALVLGGVDV      105
Beta3       VQTVAQRLKFRLNLYELK-EGRQIKPYTLMSMVANLL--YEKRF--GPYYTEPVIAGLDP      114
Beta4       TVQFAEYIQKNVQLYKMR-NGYELSPTAAANFTRRNLADCLRSR--TPYHVNLLLAGYDE      109
Beta5       CSFWERLLARQCRIYELR-NKERISVAAASKLLANMV--YQ-YKG-MGLSMGTMICGWDK      106
Beta6       CLTLTKIIEARLKMYKHS-NNKAMTTGAIAAMLSTIL--YSRRF--FPYYVYNIIGGLDE      115
Beta7       FQYLKQVLGQMVIDEELLGDGHSYSPRAIHSWLTRAM--YSRRSKMNPLWNTMVIGGYAD      117
                   :       .           :                         :: *

Beta1       QEG-GQVYSVPMGGMM-VRQSFAIGGSGSSYIYGYVDAT--YREGMTK------------      149
Beta2       -TG-PHLYSIYPHGST-DKLPYVTMGSGSLAAMAVFEDK--FRPDMEE------------      148
Beta3       KTFKPFICSLDLIGCPMVTDDFVVSGTCAEQMYGMCESL--WEPNMDP------------      160
Beta4       HEG-PALYYMDYLAAL-AKAPFAAHGYGAFLTLSILDRY--YTPTISR------------      153
Beta5       -RG-PGLYYVDSEGNR-ISGATFSVGSGSVYAYGVMDRG--YSYDLEV------------      149
Beta6       EGKGA-VYSFDPVGSY-QRDSFKAGGSAS--------AM--LQPLLDNQVGFKNMQNVEH      163
Beta7       -GE-SFLGYVDMLGVA-YEAPSLATGYGAYLAQPLLREVLEKQPVLSQ------------      162
                  :  .   .           *  :                :

Beta1       -----EECLQFTANALALAMERDGSSGGVIRLAAIAESGVERQV----------------      188
Beta2       -----EEAKNLVSEAIAAGIFNDLGSGSNIDLCVISKNKLDFLRPYTVPNKKGTRLGRYR      203
Beta3       -----DHLFETISQAMLNAVDRDAVSGMGVIVHIIEKDKITTRTL-KARMD---------      205
Beta4       -----ERAVELLRKCLEELQKRFILNLPTFSVRIIDKNGIHDLDNISFPKQGS-------      201
Beta5       -----EQAYDLARRAIYQATYRDAYSGGAVNLYHVREDGWIRVSSDNVADLHEKYSGSTP      204
Beta6       VPLSLDRAMRLVKDVFISAAERDVYTGDALRICIVTKEGIREETV-SLRKD---------      213
Beta7       -----TEARDLVERCMRVLYYRDARSYNRFQIATVTEKGVEIEGPLSTETNWD-------      210
                  .        :     .   .   . :  : :.

Beta1       -------LLGDQIPKFAVATLPPA-------    205
Beta2       CEKGTTAVLTEKITPLEIEVLEETVQTMDTS    234
Beta3       -------------------------------    205
Beta4       -------------------------------    201
Beta5       -------------------------------    204
Beta6       -------------------------------    213
Beta7       --------IAHMISGFE--------------    219
```

Figure S2. Alignment of the amino acid sequences of all seven β subunits of 20S Proteasome from *Homo Sapiens*. All conserved amino acid residues are highlighted by (*) star symbol. Polar amino acids are highlighted in green, non-polar in red, acidic in blue and basic in pink.

```
Alpha1      MSRGSSAGFDRHITIFSPEGRLYQVEYAFKAINQGGLTSVAVRGKDCAVIVTQKKVPDKL      60
Alpha2      ---MAERGYSFSLTTFSPSGKLVQIEYALAAVAGG-APSVGIKAANGVVLATEKKQKSIL      56
Alpha3      ----MSRRYDSRTTIFSPEGRLYQVEYAMEAIGHA-GTCLGILANDGVLLAAERRNIHKL      55
Alpha4      ------MSYDRAITVFSPDGHLFQVEYAQEAVKKG-STAVGVRGRDIVVLGVEKKSVAKL      53
Alpha5      -MFLTRSEYDRGVNTFSPEGRLFQVEYAIEAIKLG-STAIGIQTSEGVCLAVEKRITSPL      58
Alpha6      ---MFRNQYDNDVTVWSPQGRIHQIEYAMEAVKQG-SATVGLKSKTHAVLVALKRAQSEL      56
Alpha7      -MSSIGTGYDLSASTFSPDGRVFQVEYAMKAVENS-STAIGIRCKDGVVFGVEKLVLSKL      58
                    :.   . :**.*:: *:***  *:  .    :.:     . : . :     *

Alpha1      LDSSTV-THLFKITENIGCVMTGMTADSRSQVQRARYEAANWKYKYGYEIPVDMLCKRIA      119
Alpha2      YDERSV-HKVEPITKHIGLVYSGMGPDYRVLVHRARKLAQQYYLVYQEPIPTAQLVQRVA      115
Alpha3      LDEVFFSEKIYKLNEDMACSVAGITSDANVLTNELRLIAQRYLLQYQEPIPCEQLVTALC      115
Alpha4      QDERTV-RKICALDDNVCMAFAGLTADARIVINRARVECQSHRLTVEDPVTVEYITRYIA      112
Alpha5      MEPSSI-EKIVEIDAHIGCAMSGLIADAKTLIDKARVETQNHWFTYNETMTVESVTQAVS      117
Alpha6      AAH---QKKILHVDNHIGISIAGLTADARLLCNFMRQECLDSRFVFDRPLPVSRLVSLIG      113
Alpha7      YEEGSN-KRLFNVDRHVGMAVAGLLADARSLADIAREEASNFRSNFGYNIPLKHLADRVA      117
                    ::  :  .:    :*:  * .   .  *             :    :   :

Alpha1      DISQVYTQN-----AEMRPLGCCMILIGIDEEQGPQVYKCDPAGYYCGFKATAAGVKQTE      174
Alpha2      SVMQEYTQS-----GGVRPFGVSLLICGWN-EGRPYLFQSDPSGAYFAWKATAMGKNYVN      169
Alpha3      DIKQAYTQF-----GGKRPFGVSLLYIGWDKHYGFQLYQSDPSGNYGGWKATCIGNNSAA      170
Alpha4      SLKQRYTQS-----NGRRPFGISALIVGFDFDGTPRLYQTDPSGTYHAWKANAIGRGAKS      167
Alpha5      NLALQFGEEDADPGAMSRPFGVALLFGGVD-EKGPQLFHMDPSGTFVQCDARAIGSASEG      176
Alpha6      SKTQIPTQR-----YGRRPYGVGLLIAGYD-DMGPHIFQTCPSANYFDCRAMSIGARSQS      167
Alpha7      MYVHAYTLY-----SAVRPFGCSFMLGSYSVNDGAQLYMIDPSGVSYGYWGCAIGKARQA      172
                             ** *   :  . . .    ::   *:.      . . *

Alpha1      STSFLEKKVKKKFDWTFEQTVETAITCLSTVLS--IDFKPSEIEVGVVTV-ENPK--FRI      229
Alpha2      GKTFLEKRYNEDL--ELEDAIHTAILTLKESFE--GQMTEDNIEVGI-CN-EAGFR---R      220
Alpha3      AVSMLKQDYKEGE-MTLKSALALAIKVLNKTMDV-SKLSAEKVEIATLTR-ENGKTVIRV      227
Alpha4      VREFLEKNYTDEA--IETDDLTIKLV-IKALLEV-VQSGGKNIELAVMRR-DQSLK---I      219
Alpha5      AQSSLQEVYHKSM--TLKEAIKSSLIILKQVME--EKLNATNIELATVQP-GQNFH---M      228
Alpha6      ARTYLERHMSEFMECNLNELVKHGLRALRETLPAEQDLTTKNVSIGIVGK-DLEFT---I      223
Alpha7      AKTEIEKLQMKEM--TCRDIVKEVAKIIYIVHDE-VKDKAFELELSWVGELTNGRH---E      226
                ::.   .       .  :      :        .    ::.:.

Alpha1      LTEAEIDAHLVALAERD-----------------------      246
Alpha2      LTPTEVKDYLAAIA--------------------------      234
Alpha3      LKQKEVEQLIKKHEEEEAKAEREKKEKEQ--KEKDK----      261
Alpha4      LNPEEIEKYVAEIEKEKEENEKKKQKKAS-----------      248
Alpha5      FTKEELEEVIKDI---------------------------      241
Alpha6      YDDDDVSPFLEGLEERPQRKAQPAQPADEPAEKADEPMEH      263
Alpha7      IVPK-------DIREEAEKYAKESLKEED--ESDDDNM--      255
```

Figure S3. Alignment of the amino acid sequences of all seven α subunits of 20S Proteasome from *Homo Sapiens*. All conserved amino acid residues are highlighted by (*) star symbol. Polar amino acids are highlighted in green, non-polar in red, acidic in blue and basic in pink.

Sequence analysis was done employing Clustal Omega(1.2.4) software and used for multiple sequence alignment[1] and structural overlay of α and β subunits was obtained using a Dali server[2] to compare α1-7 and β2-7 proteins that build human 20S proteasome macromolecules. The results of this analysis are shown below.

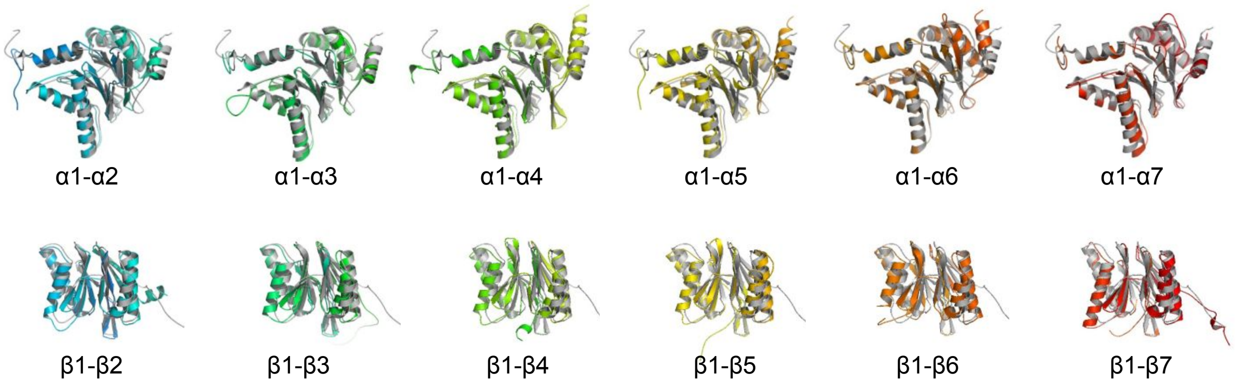

Figure S4. Alignment of the secondary structures of seven α-type and seven β-type subunits involved in the formation of 20S proteasome particle. Structures of α2-7 were overlaid on the α1 pattern, while β2-7 on the β1 pattern.

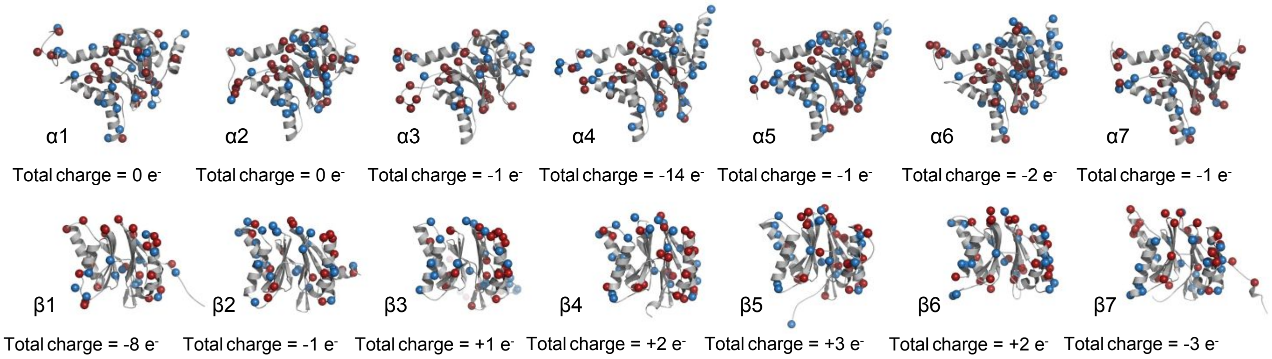

Figure S5. Secondary structures of seven α-type and seven β-type subunits with the indicated positions of charged titratable residues. Positions of Cα of Arg, Lys, and positively charged His residues are indicated by blue spheres, while those of negatively charged Glu and Asp residues are by red spheres.

1.2 Contribution of 20S Proteasome subunits in V_{elec} generated in each active site

Table S1. The contribution to the overall value of V_{elec} generated in active sites of β1, β2 and β5-subunits by a specific 20S proteasome α and β subunits located within 20Å from the bound substrate.

**β1 active site**		**β2 active site**		**β5 active site**	
Subunit	V_{elec} (kJ/mol·e$^-$)	Subunit	V_{elec} (kJ/mol·e$^-$)	Subunit	V_{elec} (kJ/mol·e$^-$)
α1	95.3 ± 0.7	α2	-19.9 ± 24.6	α5	-52.5 ± 0.7
β2	55.7 ± 4.6	β2	-534.8 ± 29.6	β4	65.6 ± 1.3
β7	89.1 ± 2.1	β3	-210.5 ± 5.3	β5	-194.5 ± 27.6
β1	-233.4 ± 32.5	β1	6.5 ± 0.9	β6	146.0 ± 4.8
β6'	106.0 ± 0.7	β5'	1.6 ± 0.3	β3'	22.9 ± 3.9
β7'	80.7 ± 4.6	β6'	-87.5 ± 6.1	β4'	107.9 ± 1.7
β1'	-4.5 ± 1.4	β7'	82.9 ± 1.4		

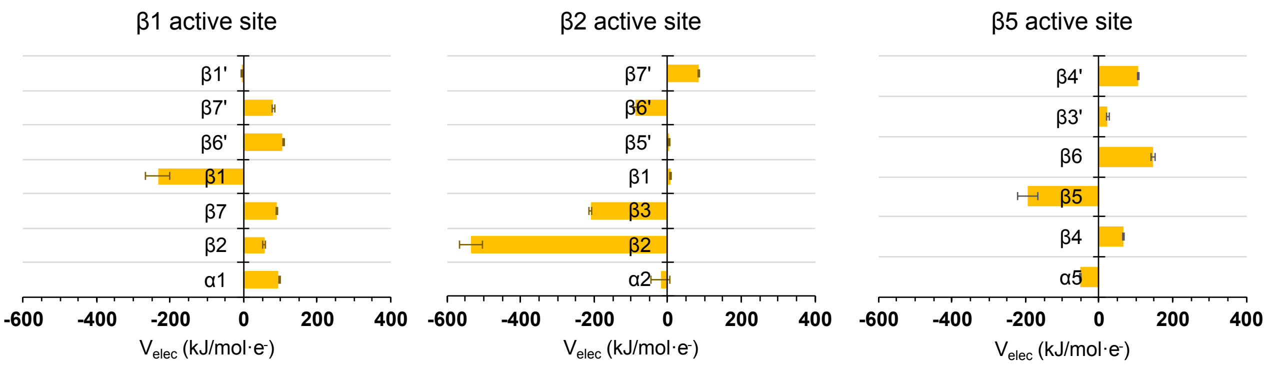

Figure S6. Graphical representation of the contribution to the overall value of V_{elec} generated in active sites of β1, β2 and β5 subunits by a specific 20S proteasome α and β subunits located within 20Å from the bound substrate.

2. Mechanism of reaction in three catalytically active subunits of the 20S proteasome.

2.1 Free energy surfaces (FESs) computed for β1-subunit

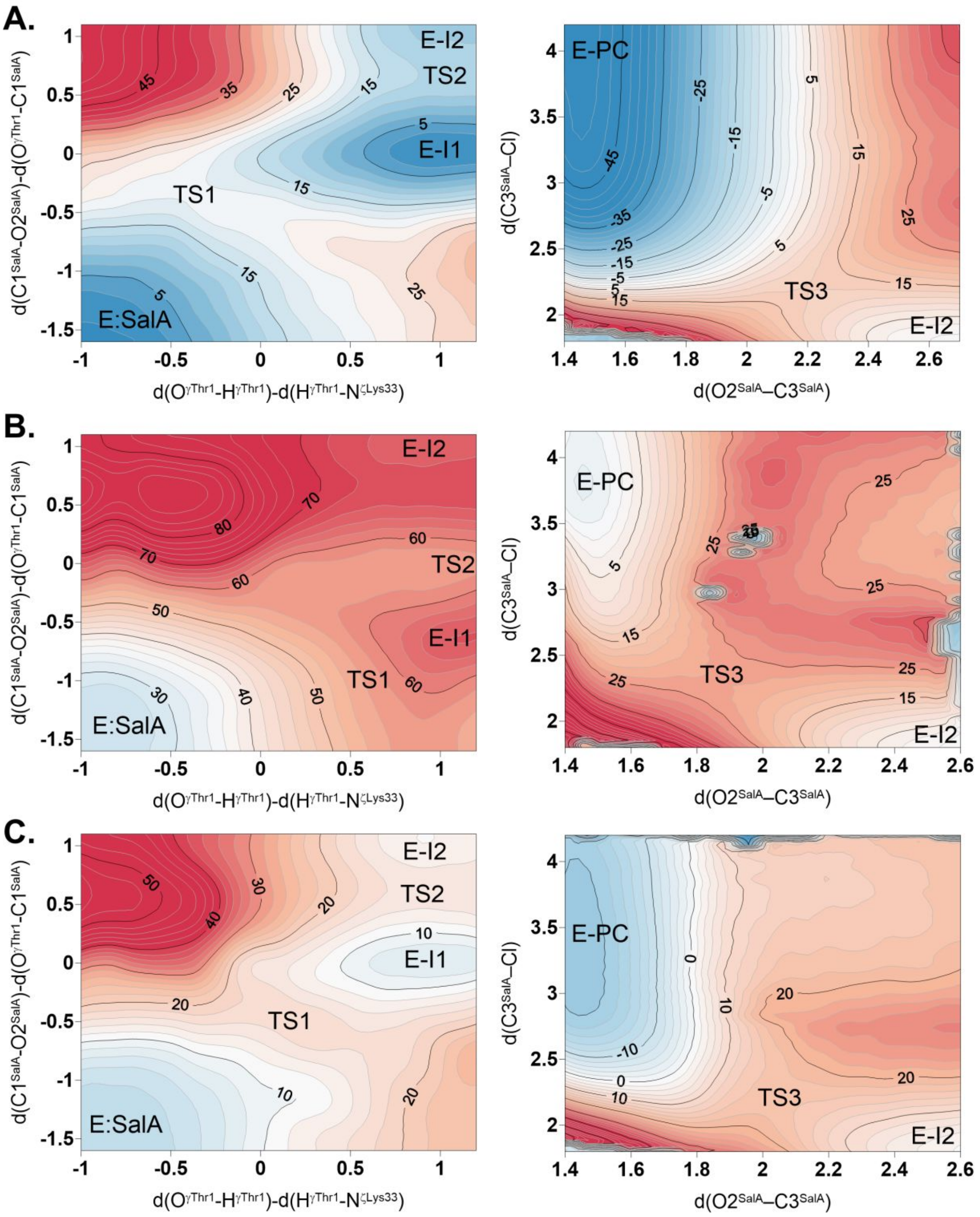

Figure S7. Free energy surfaces (FESs) computed at M06-2X:AM1/AMBER level for inactivation of β1 subunit with SalA in **A.** unperturbed (V_{elec}(ON)) enzyme, **B.** electrostatically neutral variant (V_{elec}(OFF)) and **C.** electrostatically active Asp17 (V_{elec}(D17)). Values of energy are in kcal/mol.

2.2 Free energy surfaces (FESs) computed for β2-subunit

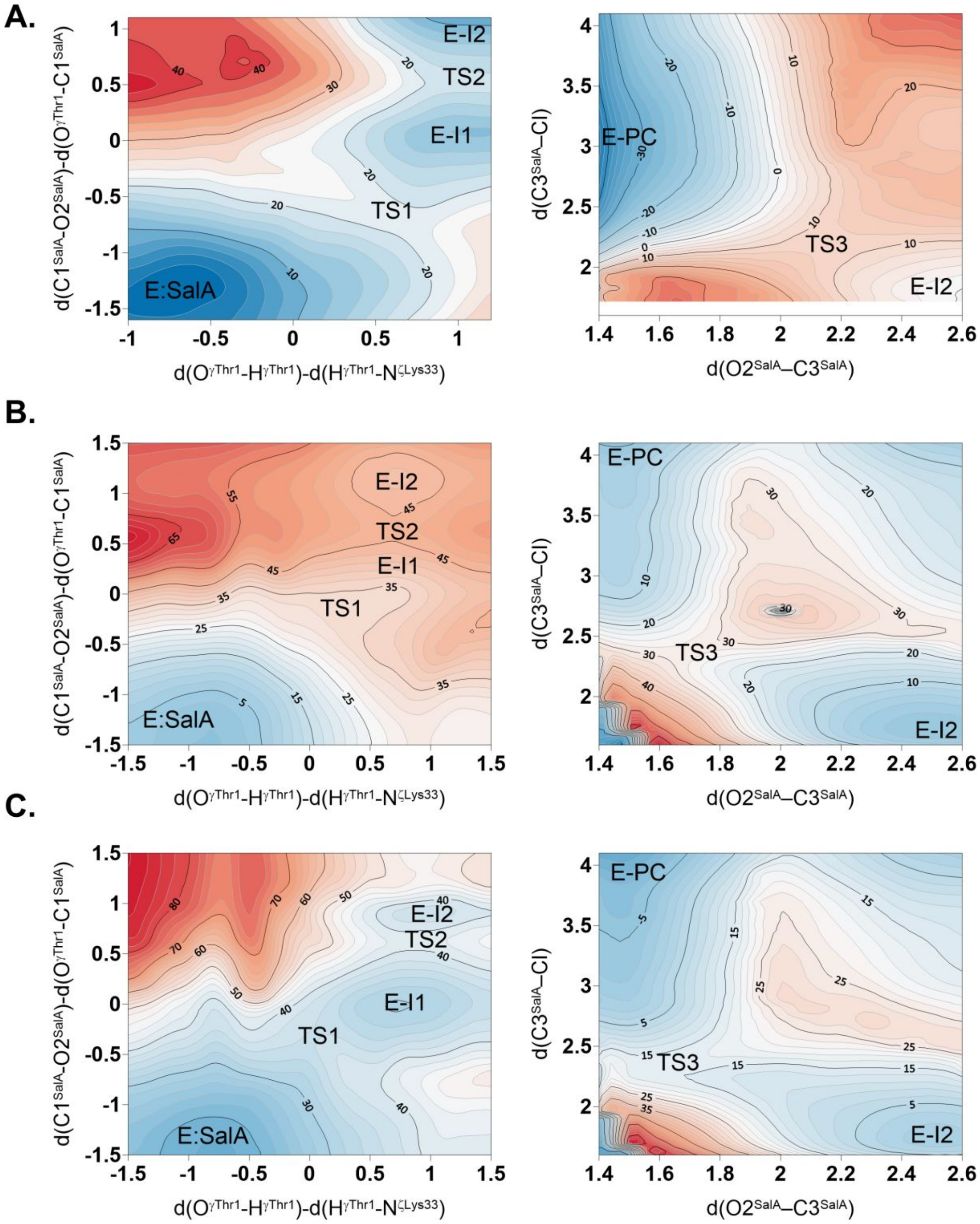

Figure S8. Free energy surfaces (FESs) computed at M06-2X:AM1/AMBER level for inactivation of β2 subunit with SalA in **A.** unperturbed (V_{elec}(ON)) enzyme, **B.** electrostatically neutral variant (V_{elec}(OFF)) and **C.** electrostatically active Asp17 (V_{elec}(D17)). Values of energy are in kcal/mol.

2.3 Free energy surfaces (FESs) computed for β5-subunit

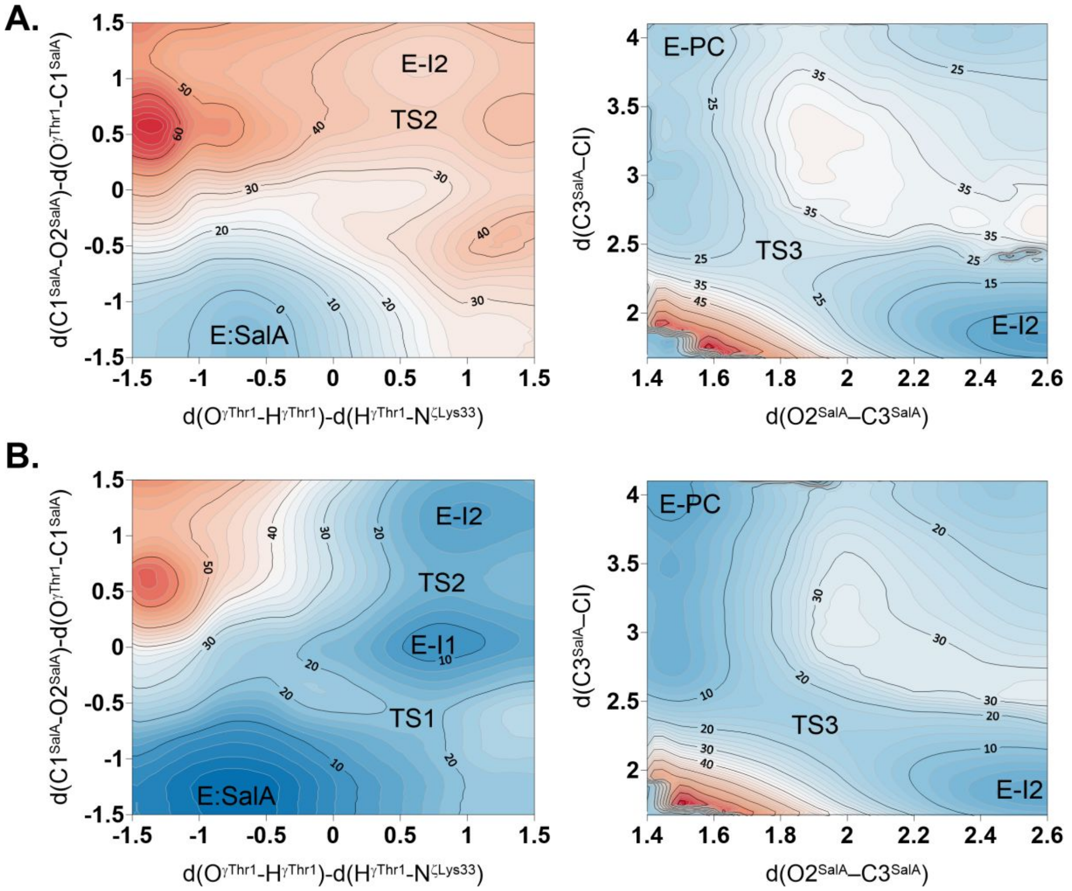

Figure S9. Free energy surfaces (FESs) computed at M06-2X:AM1/AMBER level for inactivation of β5 subunit with SalA in **A.** electrostatically neutral variant (V_{elec}(OFF)) and **B.** electrostatically active Asp17 (V_{elec}(D17)). Note that: FESs for inactivation of β5 in unperturbed (V_{elec}(ON)) enzyme were computed previously (see reference 1a). Values of energy are in kcal/mol.

3. The hydration shell of the chloride in the E-I2 and E-PC structures.

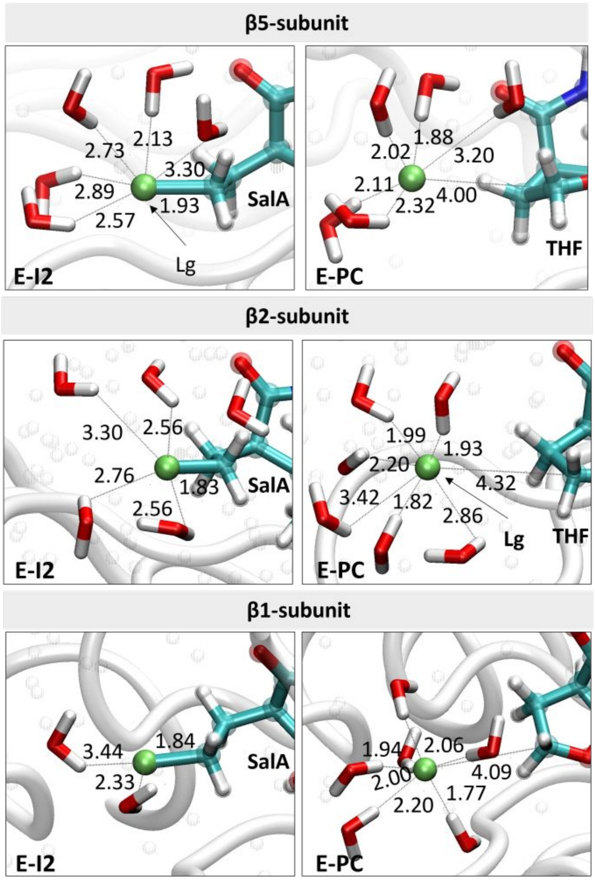

Figure S10. Snapshots structures representing first hydration shell of the chloride in the E-I2 and E-PC structures in three catalytically active β-subunits. All water molecules within 3.5 Å from chloride ion (leaving group, Lg) are highlighted.

4. Contribution of individual amino acid residues in electrostatic potential (V_{elec}) generated on the electrophile in the E:SalA complex

4.1 Electrostatic potential (V_{elec}) in β5-subunit

Table S2. Electrostatic potential (V_{elec}) generated in the active site of the β5 subunit by each residue located within 20 Å. Distances collected in the table correspond to the distance between the C1 carbon atom of SalA and Cα of each amino acid residue.

Subunit	Residue	V_{elec} (kJ/mol·e$^-$)	Distance (Å)	Catalytic Subunit	Residue	V_{elec} (kJ/mol·e$^-$)	Distance (Å)
α5	GLN89	-0.86 ± 0.18	28.7	β5	THR3	4.80 ± 2.34	9.9
	TRP92	-0.84 ± 0.18	26.3		LEU4	-3.12 ± 0.67	13.7
	PHE93	-1.01 ± 0.19	23.7		ALA5	-0.97 ± 0.29	16.6
	THR94	-0.56 ± 0.28	24.9		PHE6	-0.97 ± 0.27	20.3
	TYR95	-0.38 ± 0.21	25.6		LYS7	55.42 ± 0.50	23.6
	ASN96	-0.23 ± 0.43	24.0		PHE8	-0.30 ± 0.13	26.5
	GLU97	-48.58 ± 0.37	27.7		VAL12	0.65 ± 0.24	23.0
					ILE13	-0.19 ± 0.23	20.8
β4	MET1	54.08 ± 0.42	23.9		VAL14	2.56 ± 0.48	17.0
	VAL20	0.69 ± 0.09	24.5		ALA15	0.44 ± 0.67	14.6
	ALA21	0.50 ± 0.20	21.1		ALA16	15.95 ± 1.30	11.2
	ALA22	0.15 ± 0.32	18.2		ASP17	-426.26 ± 20.70	10.1
	SER23	-0.19± 0.51	14.7		SER18	27.89 ± 5.10	9.6
	ASN24	7.96 ± 0.56	13.8		ARG19	128.03 ± 9.34	7.8
	ILE25	1.76 ± 0.24	16.9		ALA20	-3.29 ± 1.85	6.9
	VAL26	-0.56 ± 0.29	17.6		THR21	-11.82 ± 1.37	8.3
	GLN27	-1.66 ± 0.33	19.7		ALA22	1.75 ± 0.59	10.9
	MET28	-1.57 ± 0.43	18.8		GLY23	-1.27 ± 1.16	12.0
	LYS29	54.61 ± 0.40	22.1		ALA24	-3.38 ± 0.73	13.2
	GLU49	-50.92 ± 0.33	21.2		TYR25	-4.62 ± 0.64	13.8
	ALA50	-1.91 ± 0.13	21.0		ILE26	5.30 ± 0.84	11.8
	GLY51	0.37 ± 0.08	21.3		ALA27	5.33 ± 0.80	12.0
	GLN55	2.26 ± 0.20	25.7		SER28	-9.20 ± 2.14	11.6
					GLN29	-13.31 ± 2.12	12.2
β6	ARG1	87.55 ± 0.49	30.9		THR30	2.57 ± 1.04	13.2
	TYR5	-0.47 ± 0.22	23.6		VAL31	-9.84 ± 6.11	10.7
	PHE7	0.97 ± 0.13	23.6		LYS32	107.90 ± 3.06	11.0
	ILE12	-0.90 ± 0.16	24.6		VAL34	-5.05 ± 1.18	12.4
	LEU13	1.23 ± 0.11	24.5		ILE35	-0.93 ± 0.43	14.8
	ALA14	-0.78 ± 0.17	24.5		GLU36	-91.72 ± 1.26	18.6
	ALA16	-0.73 ± 0.12	27.1		ILE37	0.59 ± 0.17	20.0
	ILE52	1.33 ± 0.06	26.1		ASN38	-2.88 ± 0.21	23.8
	GLY53	-1.14 ± 0.11	25.0		PRO39	0.17 ± 0.15	26.4
	CYS54	1.51 ± 0.18	23.6		TYR40	0.39 ± 0.20	25.6
	SER55	-1.19 ± 0.28	23.0		LEU41	0.06 ± 0.20	22.0
	GLY56	0.69 ± 0.09	22.2		LEU42	2.37 ± 0.36	18.5
	PHE57	-0.55 ± 0.10	24.4		GLY43	-0.67 ± 0.42	15.1
	ASP60	-51.03 ± 0.34	25.4		THR44	2.44 ± 0.85	11.6
	LEU64	0.84 ± 0.12	25.9		MET45	-11.75 ± 1.28	8.4
	ILE68	0.50 ± 0.08	28.1		ALA46	10.40 ± 0.97	5.4
	THR85	-0.38 ± 0.13	27.5		GLY47	-9.24 ± 1.03	5.2
	GLY86	-1.42 ± 0.11	25.0		GLY48	0.20 ± 0.89	6.9
	ALA87	-1.32 ± 0.17	25.8		ALA49	4.50 ± 0.92	7.3
	ILE88	-0.87 ± 0.11	25.7		ALA50	1.30 ± 0.50	11.0
	ALA89	-1.94 ± 0.09	22.2		ASP51	-87.55 ± 1.07	11.7
	ALA90	-1.20 ± 0.16	21.1		CYS52	4.70 ± 1.00	10.6
	MET91	0.27 ± 0.20	23.6		SER53	-0.21 ± 0.75	12.8
	LEU92	0.86 ± 0.25	21.5		PHE54	1.34 ± 0.27	15.6
	SER93	-0.36 ± 0.57	18.6		TRP55	3.59 ± 0.24	16.1
	THR94	1.35 ± 0.20	21.2		GLH56	4.75 ± 0.37	15.8
	ILE95	0.77 ± 0.13	22.7		ARG57	74.34 ± 0.92	18.2
	LEU96	1.63 ± 0.23	19.5		LEU58	1.50 ± 0.12	20.4

	TYR97	2.06 ± 0.25	18.9		LEU59	2.44 ± 0.13	20.7
	SER98	1.22 ± 0.13	22.6		ALA60	1.94 ± 0.12	21.6
	ARG99	52.37 ± 0.39	21.6		ARG61	53.34 ± 0.41	24.1
	ARG100	76.61 ± 0.88	18.1		GLN62	0.80 ± 0.15	25.8
	PHE101	0.09 ± 0.20	19.0		CYS63	1.33 ± 0.18	25.9
	PHE102	0.63 ± 0.16	21.2		ARG64	46.49 ± 0.24	28.2
	PRO103	2.21 ± 0.14	19.1		VAL76	-0.66 ± 0.15	24.6
	TYR104	-1.95 ± 0.16	20.5		ALA79	0.03 ± 0.21	23.4
	TYR105	2.12 ± 0.19	19.7		SER80	-0.15 ± 0.24	21.1
	VAL106	-1.31 ± 0.19	19.8		LYS81	45.18 ± 0.32	23.9
	TYR107	4.03 ± 0.68	17.9		LEU82	0.19 ± 0.12	24.0
	ASN108	-0.98 ± 0.43	19.5		LEU83	0.86 ± 0.23	20.2
	ILE109	1.14 ± 0.13	20.6		ALA84	-0.08 ± 0.19	20.4
	ILE110	-1.32 ± 0.13	23.1		ASN85	-0.12 ± 0.21	23.5
	GLY111	0.21 ± 0.09	25.7		MET86	0.26 ± 0.23	22.0
	ALA120	1.34 ± 0.10	26.0		VAL87	0.81 ± 0.19	18.9
	VAL121	-0.74 ± 0.19	23.0		TYR88	-0.06 ± 0.14	21.4
	TYR122	2.98 ± 0.30	19.7		GLN89	-0.15 ± 0.15	24.0
	SER123	1.93 ± 0.60	17.0		TYR90	-0.92 ± 0.24	21.6
	PHE124	0.25 ± 0.55	15.7		LYS91	57.23 ± 0.38	21.0
	ASP125	-96.43 ± 1.55	14.3		GLY92	1.09 ± 0.09	22.1
	PRO126	1.79 ± 0.18	15.8		MET93	0.94 ± 0.29	19.8
	VAL127	2.38 ± 0.34	14.8		GLY94	-1.04 ± 0.39	16.4
	GLY128	-0.23 ± 0.53	14.4		LEU95	2.09 ± 0.19	13.5
	SER129	2.50 ± 1.01	11.4		SER96	-2.66 ± 0.44	10.6
	TYR130	-4.87 ± 0.94	13.5		MET97	5.79 ± 0.38	9.0
	GLN131	-8.14 ± 1.33	14.6		GLY98	-5.12 ± 0.55	10.2
	ARG132	62.99 ± 0.62	18.0		THR99	5.05 ± 0.44	11.5
	ASP133	-95.13 ± 1.87	19.0		MET100	-3.36 ± 0.73	14.4
	SER134	0.52 ± 0.24	22.4		ILE101	-0.23 ± 0.30	17.4
	PHE135	-1.48 ± 0.34	22.7		CYS102	-1.19 ± 0.43	19.6
	LYS136	98.92 ± 1.78	20.4		GLY103	-0.48 ± 0.16	23.1
	ALA137	-2.54 ± 0.15	20.9		TRP104	-1.00 ± 0.12	26.8
	GLY138	1.30 ± 0.14	19.6		GLY110	-1.00 ± 0.13	24.4
	GLY139	-1.54 ± 0.18	21.0		LEU111	2.04 ± 0.20	21.0
	SER140	-1.81 ± 0.46	22.4		TYR112	-1.69 ± 0.26	19.5
	ALA141	0.08 ± 0.16	23.4		TYR113	1.36 ± 0.22	16.4
	SER142	1.87 ± 0.55	20.8		VAL114	-3.01 ± 0.16	16.0
	ALA143	-0.40 ± 0.23	20.3		ASP115	-79.06 ± 1.11	14.4
	MET144	-0.36 ± 0.27	24.0		SER116	2.59 ± 0.68	14.2
	LEU145	0.77 ± 0.16	24.0		GLU117	-61.37± 0.72	17.2
	GLN146	1.03 ± 0.77	20.9		GLY118	1.55 ± 0.20	19.5
	PRO147	0.07 ± 0.15	21.9		ASN119	0.32 ± 0.30	19.4
	LEU148	0.13 ± 0.17	25.5		ARG120	55.73 ± 0.35	20.0
	LEU149	1.23 ± 0.18	24.5		ILE121	-1.56 ± 0.11	21.2
	ASP150	-81.95 ± 0.84	22.6		SER122	-0.75 ± 0.20	22.6
	ASN151	1.54 ± 0.20	25.8		GLY123	0.54 ± 0.27	22.2
	GLY154	0.29 ± 0.19	24.4		ALA124	-0.26 ± 0.21	23.1
	LYS156	86.61 ± 1.62	24.2		THR125	1.85 ± 0.34	20.0
	ASN157	-1.18 ± 0.22	26.5		PHE126	-0.60 ± 0.40	16.4
					SER127	5.90 ± 0.61	13.2
β3’	MET1	61.88 ± 0.57	19.9		VAL128	-6.75 ± 1.18	9.8
	SER2	-1.62 ± 0.15	21.8		GLY129	11.84 ± 1.01	6.7
	ILE3	-1.16 ± 0.16	22.0		SER130	2.21 ± 1.31	7.4
	MET4	-0.14 ± 0.19	22.9		GLY131	10.78 ± 0.85	9.8
	SER5	-0.17 ± 0.20	19.3		SER132	9.82 ± 0.89	11.3
	TYR6	-1.39 ± 0.34	18.7		VAL133	2.82 ± 0.35	13.9
	ASN7	1.16 ± 0.53	19.7		TYR134	3.91 ± 0.41	15.4
	GLY8	1.90 ± 0.36	19.1		ALA135	4.72 ± 0.26	15.6
	GLY9	0.10 ± 0.25	21.5		TYR136	0.89 ± 0.30	17.2
	ALA10	-0.27 ± 0.13	23.4		GLY137	2.37 ± 0.14	19.9
	ASP25	-62.64 ± 0.48	24.3		VAL138	2.62 ± 0.17	21.2
	ARG26	60.52 ± 0.51	24.3		MET139	0.00 ± 0.24	20.5
	ARG27	101.02 ± 1.26	21.0		ASP140	-63.56± 0.74	23.4

	PHE28	-0.87 ± 0.30	21.3
	GLY29	2.35 ± 0.25	18.6
	ILE30	-2.14 ± 0.25	17.6
	GLN31	-3.37 ± 0.61	15.0
	ALA32	-4.40 ± 0.32	11.9
	GLN33	1.96 ± 0.99	13.8
	MET34	1.52 ± 0.29	16.7
	VAL35	0.25 ± 0.22	20.4
	THR36	-1.39 ± 0.25	22.7
	THR37	0.12 ± 0.19	23.2
	LYS41	61.02 ± 0.46	29.4
	GLY55	-0.64 ± 0.21	24.6
	LEU56	0.84 ± 0.08	24.6
	GLY140	0.80 ± 0.13	23.4
	THR141	0.69 ± 0.42	19.9
	CYS142	-0.25 ± 0.75	20.2
	ALA143	0.34 ± 0.13	23.6
	GLU144	-63.19 ± 0.72	23.0
	GLN145	4.21 ± 0.47	22.0
	MET146	-0.02 ± 0.19	24.9
	MET171	-1.44 ± 0.18	25.6
	LEU172	-2.65 ± 0.11	23.8
	ASN173	0.06 ± 0.26	22.7
	ALA174	-2.76 ± 0.24	21.5
	VAL175	-3.48 ± 0.22	19.4
	ASH176	-4.64 ± 0.56	17.8
	ARG177	99.59 ± 1.83	16.1
	ASP178	-85.33 ± 1.36	15.5
	ALA179	-0.48 ± 0.52	12.7
	VAL180	0.70 ± 0.30	15.3
	SER181	-1.84 ± 0.25	18.5
	GLY182	1.56 ± 0.15	19.7
	MET183	4.27 ± 0.25	20.2
	GLY184	-0.78 ± 0.17	23.4
	ALA202	0.85 ± 0.18	23.9
	ARG203	107.17 ± 1.37	20.3
	MET204	-2.23 ± 0.43	18.1
	ASP205	-242.67 ± 3.66	14.5
β4’	TYR3	1.63 ± 0.09	27.7
	TYR117	0.83 ± 0.23	28.3
	LEU121	0.82 ± 0.10	27.0
	PHE129	-0.31 ± 0.12	28.5
	ALA131	-0.66 ± 0.18	25.7
	GLY133	-0.64 ± 0.14	23.8
	TYR134	-1.17 ± 0.22	20.5
	GLY135	-1.25 ± 0.25	22.0
	ALA136	-0.55 ± 0.24	21.4
	PHE137	-2.19 ± 0.53	17.7
	LEU138	-0.52 ± 0.28	19.1
	THR139	0.83 ± 0.31	22.3
	LEU140	1.24 ± 0.23	21.9
	SER141	2.82 ± 0.33	20.4
	ILE142	0.37 ± 0.21	23.8
	LEU143	0.95 ± 0.12	25.6
	ASP144	-70.65 ± 0.91	23.6
	ARG145	59.67 ± 0.54	25.0
	GLH166	0.33 ± 0.19	25.5
	LEU167	-0.16 ± 0.14	25.8
	LYS169	48.58 ± 0.41	25.9
	ARG170	65.69 ± 0.72	22.2
	PHE171	1.22 ± 0.11	22.1
	ILE172	1.52 ± 0.09	22.0
	LEU173	-0.52 ± 0.09	24.8

	ARG141	53.97 ± 0.49	25.4
	GLY142	-0.54 ± 0.29	25.6
	TYR143	0.60 ± 0.26	25.8
	ALA152	-2.09 ± 0.11	24.9
	TYR153	-3.18 ± 0.26	23.1
	ASP154	-68.26 ± 0.79	23.6
	LEU155	-2.51 ± 0.23	22.1
	ALA156	-3.68 ± 0.49	18.9
	ARG157	68.87 ± 0.99	19.0
	ARG158	66.86 ± 0.78	19.7
	ALA159	-1.76 ± 0.48	17.0
	ILE160	-2.09 ± 0.79	14.1
	TYR161	-2.59 ± 0.67	16.3
	GLN162	2.59 ± 0.49	16.9
	ALA163	3.35 ± 0.96	13.2
	THR164	2.62 ± 0.86	12.8
	TYR165	2.38 ± 0.28	16.2
	ARG166	81.51 ± 1.09	14.8
	ASP167	-151.87 ± 5.51	11.3
	ALA168	-3.79 ± 1.19	10.4
	TYR169	-33.87 ± 5.36	6.6
	SER170	30.19 ± 5.74	7.9
	GLY171	-10.87 ± 2.50	10.6
	GLY172	3.36 ± 0.90	12.9
	ALA173	-10.20 ± 1.18	14.6
	VAL174	2.92 ± 0.67	14.9
	ASN175	2.80 ± 0.94	15.6
	LEU176	0.12 ± 0.36	17.7
	TYR177	-1.37 ± 0.61	19.8
	HIE178	-2.69 ± 0.34	22.6
	VAL179	-0.20 ± 0.16	23.9
	TRP184	0.83 ± 0.30	24.5
	ILE185	-0.71 ± 0.26	24.2
	ARG186	89.41 ± 1.25	21.6
	VAL187	1.89 ± 0.18	23.3
	SER188	-0.25 ± 0.50	21.4
	SER189	2.17 ± 0.65	19.5
	ASP190	-85.74 ± 1.20	19.9
	ASN191	0.36 ± 0.80	19.1
	VAL192	2.91 ± 0.38	19.2
	ALA193	1.58 ± 0.22	22.0
	ASP194	-61.15 ± 0.46	24.2
	LEU195	2.60 ± 0.33	22.3
	HIE196	2.09 ± 0.52	22.4
	GLU197	-55.54 ± 0.39	26.2
	TYR199	-1.10 ± 0.29	24.9

4.2 Electrostatic potential (V_{elec}) in β2-subunit.

Table S3. Electrostatic potential (V_{elec}) generated in the active site of the β2 subunit by each residue located within 20 Å.

Subunit	Residue	V_{elec} (kJ/mol·e$^-$)	Distance (Å)	Catalytic Subunit	Residue	V_{elec} (kJ/mol·e$^-$)	Distance (Å)
α2	GLN95	-0.88 ± 1.34	27.5	β2	THR2	0.59 ± 3.80	7.6
	TYR98	-0.20 ± 0.46	25.3		ILE3	-0.69 ± 0.97	9.5
	LEU99	-0.19 ± 0.50	22.9		ALA4	-3.71 ± 0.49	13.3
	VAL100	-0.32 ± 0.70	23.6		GLY5	-1.16 ± 0.25	16.7
	TYR101	-0.10 ± 0.40	24.5		VAL6	-1.20 ± 0.18	20.4
	GLN102	-0.75 ± 0.99	22.7		ILE12	1.14 ± 0.27	22.7
	GLU103	-17.42 ± 24.54	26.2		VAL13	0.79 ± 0.27	20.3
					LEU14	2.97 ± 0.49	16.5
β3	MET1	58.36 ± 0.57	22.2		GLY15	1.29 ± 0.84	14.0
	SER2	1.57 ± 0.21	21.9		ALA16	18.54 ± 2.72	10.6
	ILE3	0.78 ± 0.12	23.4		ASP17	-415.56 ± 20.64	9.7
	MET4	0.74 ± 0.37	21.6		THR18	22.66 ± 7.00	9.6
	SER5	0.57 ± 0.12	23.7		ARG19	90.23 ± 8.87	8.0
	ALA10	1.38 ± 0.05	24.6		ALA20	-0.72 ± 2.37	6.6
	VAL11	-1.15 ± 0.13	24.6		THR21	-10.19 ± 2.06	7.4
	MET12	1.78 ± 0.15	24.2		GLU22	-101.34 ± 2.73	9.5
	ALA13	-1.26 ± 0.14	24.7		GLY23	-3.87 ± 1.45	11.1
	LYS15	62.27 ± 0.53	27.5		MET24	0.04 ± 0.98	12.6
	ILE51	1.33 ± 0.05	25.7		VAL25	-4.91 ± 0.61	12.7
	GLY52	-1.23 ± 0.08	24.9		VAL26	5.99 ± 0.77	11.3
	LEU53	1.13 ± 0.08	23.4		ALA27	6.94 ± 0.88	11.1
	ALA54	-1.06 ± 0.14	22.7		ASP28	-146.52 ± 4.20	11.4
	GLY55	0.64 ± 0.07	22.2		LYS29	99.45 ± 3.94	12.5
	LEU56	-0.51 ± 0.07	24.8		ASN30	8.09 ± 2.51	12.8
	VAL63	0.60 ± 0.14	25.2		CYS31	8.45 ± 7.27	9.7
	PRO84	-0.51 ± 0.20	25.8		SER32	-2.95 ± 2.98	10.7
	TYR85	-2.04 ± 0.21	23.9		LYN33	-16.52 ± 1.86	9.1
	THR86	-1.37 ± 0.24	25.5		ILE34	-2.87 ± 0.92	12.5
	LEU87	-0.32 ± 0.14	25.0		HIE35	5.05 ± 1.12	14.8
	MET88	-0.72 ± 0.44	21.2		PHE36	-1.99 ± 0.39	18.5
	SER89	-2.37 ± 0.25	20.8		ILE37	0.64 ± 0.16	20.2
	MET90	-0.06 ± 0.20	23.0		SER38	-0.83 ± 0.18	23.8
	VAL91	0.34 ± 0.20	21.3		PRO39	-0.88 ± 0.21	25.9
	ALA92	0.41 ± 0.28	17.9		ASN40	-0.72 ± 0.28	25.6
	ASN93	1.55 ± 0.26	20.0		ILE41	0.37 ± 0.18	22.1
	LEU94	0.60 ± 0.14	21.8		TYR42	1.75 ± 0.30	18.5
	LEU95	1.54 ± 0.26	18.7		CYS43	1.35 ± 0.66	15.0
	TYR96	2.01 ± 0.33	17.4		CYS44	9.64 ± 1.46	11.3
	GLU97	-51.08 ± 0.43	21.0		GLY45	-17.15 ± 1.46	8.2
	LYS98	50.36 ± 0.57	20.5		ALA46	10.77 ± 0.95	4.8
	ARG99	83.10 ± 1.90	16.9		GLY47	2.09 ± 2.23	4.6
	PHE100	-0.40 ± 0.23	18.5		THR48	-0.16 ± 1.80	5.9
	GLY101	1.38 ± 0.15	19.4		ALA49	-1.20 ± 1.17	6.3
	PRO102	1.27 ± 0.26	18.9		ALA50	-0.65 ± 0.77	10.0
	TYR103	-1.58 ± 0.16	21.3		ASP51	-94.20 ± 2.31	10.7
	TYR104	1.28 ± 0.21	20.6		THR52	10.22 ± 1.26	9.9
	THR105	-0.75 ± 0.37	19.8		ASP53	-96.65 ± 1.59	12.5
	GLH106	0.58 ± 0.51	17.4		MET54	4.29 ± 0.35	14.6
	PRO107	-3.37 ± 0.16	19.7		THR55	5.42 ± 0.27	15.2
	VAL108	1.40 ± 0.13	20.4		THR56	3.74 ± 0.59	15.9
	ILE109	-1.02 ± 0.13	22.7		GLN57	6.09 ± 0.28	18.2
	ALA110	0.44 ± 0.09	25.6		LEU58	1.75 ± 0.13	20.5
	PHE120	1.78 ± 0.15	25.0		ILE59	2.47 ± 0.10	20.9
	ILE121	-1.01 ± 0.20	22.2		SER60	3.32 ± 0.22	21.6
	CYS122	1.20 ± 0.30	18.9		SER61	1.62 ± 0.18	24.0
	SER123	-0.03 ± 0.74	16.7		ASN62	3.02 ± 0.11	25.9
	LEU124	1.74 ± 0.49	14.6		LEU63	1.78 ± 0.08	26.2
	ASP125	-110.26 ± 3.17	12.1		GLU64	-53.54 ± 0.31	27.2

	LEU126	1.70 ± 0.25	14.1		VAL76	-0.90 ± 0.11	24.2
	ILE127	2.33 ± 0.53	12.9		THR78	-0.12 ± 0.13	25.3
	GLY128	0.62 ± 0.56	14.1		ALA79	-0.36 ± 0.26	21.6
	CYS129	0.50 ± 1.40	11.8		ASN80	1.50 ± 0.20	20.9
	PRO130	-7.80 ± 0.71	13.8		ARG81	46.44 ± 0.31	23.3
	MET131	-0.90 ± 0.87	13.1		MET82	0.21 ± 0.20	22.2
	VAL132	-5.49 ± 0.56	16.3		LEU83	0.42 ± 0.25	18.5
	THR133	1.57 ± 0.54	17.9		LYS84	52.41 ± 0.48	20.1
	ASP134	-70.00 ± 0.79	21.4		GLN85	-0.15 ± 0.23	21.9
	ASP135	-78.18 ± 0.99	22.8		MET86	-0.17 ± 0.25	19.7
	PHE136	-2.44 ± 0.19	21.7		LEU87	0.93 ± 0.21	16.8
	VAL137	2.29 ± 0.12	19.6		PHE88	0.25 ± 0.20	19.7
	VAL138	-2.31 ± 0.15	19.7		ARG89	49.56 ± 0.35	21.4
	SER139	1.28 ± 0.47	19.2		TYR90	-0.69 ± 0.32	18.9
	GLY140	-1.67 ± 0.16	21.0		GLN91	1.04 ± 0.37	17.6
	THR141	-1.33 ± 0.46	22.0		GLY92	0.79 ± 0.27	17.7
	CYS142	0.21 ± 0.24	22.8		TYR93	-1.01 ± 0.23	15.6
	ALA143	0.35 ± 0.33	19.4		ILE94	2.78 ± 0.21	12.4
	GLU144	-70.31 ± 1.22	19.5		GLY95	-1.63 ± 0.82	9.6
	GLN145	-2.14± 0.23	23.2		ALA96	5.74 ± 0.43	8.6
	MET146	0.15 ± 0.26	22.6		ALA97	-5.54 ± 0.59	9.6
	TYR147	1.31 ± 0.71	20.1		LEU98	2.40 ± 0.45	11.1
	GLY148	-0.09 ± 0.21	22.7		VAL99	-2.17 ± 0.55	13.4
	MET149	0.24 ± 0.21	25.4		LEU100	-0.52 ± 0.33	16.6
	CYS150	1.66 ± 0.28	23.7		GLY101	-1.20 ± 0.28	19.7
	GLU151	-82.07 ± 0.97	22.5		GLY102	-0.55 ± 0.12	23.0
					HID109	-0.36 ± 0.15	24.3
β1	THR20	-0.33 ± 0.27	22.4		LEU110	2.16 ± 0.18	20.8
	THR21	1.30 ± 0.42	20.2		TYR111	-2.29 ± 0.20	18.9
	THR22	1.20 ± 0.31	16.9		SER112	3.37 ± 0.25	15.9
	GLY23	0.69 ± 0.19	18.8		ILE113	-3.35 ± 0.15	15.2
	SER24	1.62 ± 0.30	20.1		TYR114	3.23 ± 0.38	13.6
	TYR25	1.61 ± 0.45	18.4		PRO115	1.96 ± 0.18	13.2
	ILE26	-1.23 ± 0.26	20.1		HID116	1.28 ± 0.40	16.8
	ALA27	-0.92 ± 0.18	18.0		GLY117	1.54 ± 0.25	18.7
	ASN28	0.99 ± 0.21	21.3		SER118	-2.55 ± 0.31	19.0
	ALA49	1.02 ± 0.16	22.2		THR119	1.71 ± 0.14	19.5
	ALA50	0.60 ± 0.11	20.6		ASP120	-63.00 ± 0.57	20.4
	GLN53	-0.76 ± 0.17	24.4		LYS121	52.41 ± 0.41	21.4
	ALA54	0.72 ± 0.10	23.7		LEU122	-0.94 ± 0.18	22.3
					TYR124	1.81 ± 0.22	20.1
β5'	ALA22	-0.82 ± 0.07	27.2		VAL125	-0.97 ± 0.34	16.6
	GLY23	0.88 ± 0.08	25.6		THR126	7.63 ± 0.64	13.0
	ALA24	0.98 ± 0.14	26.1		MET127	-4.62 ± 1.36	9.8
	TYR25	0.56 ± 0.19	26.3		GLY128	10.61 ± 0.57	6.7
					SER129	2.46 ± 0.87	7.7
β6'	SER3	0.46 ± 0.14	27.1		GLY130	8.51 ± 0.76	10.2
	TYR5	0.59 ± 0.10	26.5		SER131	7.56 ± 2.58	11.0
	VAL6	1.11 ± 0.15	22.7		LEU132	1.77 ± 0.34	14.1
	PHE7	-0.91 ± 0.26	21.5		ALA133	3.79 ± 0.34	15.5
	ASN8	1.27 ± 0.34	21.0		ALA134	4.27 ± 0.32	15.1
	GLY9	2.20 ± 0.16	19.8		MET135	0.67 ± 0.34	17.0
	GLY10	0.56 ± 0.24	21.6		ALA136	2.30 ± 0.13	19.6
	THR11	0.03 ± 0.20	23.7		VAL137	3.02 ± 0.16	20.5
	ASP26	-61.39 ± 0.46	24.6		PHE138	2.50 ± 0.19	21.0
	THR27	-0.15 ± 0.23	24.1		ALA151	-2.18 ± 0.15	23.7
	ARG28	92.15 ± 1.13	20.6		LYS152	61.38 ± 0.74	22.3
	LEU29	-1.46 ± 0.27	20.3		ASN153	-5.31 ± 0.31	23.1
	SER30	2.59 ± 0.58	17.8		LEU154	-2.41 ± 0.23	21.5
	GLU31	-59.22 ± 0.52	17.7		VAL155	-3.28 ± 0.51	18.1
	GLY32	-1.53 ± 0.26	15.5		SER156	-5.41 ± 1.02	18.2
	PHE33	-3.77 ± 0.33	12.1		GLU157	-70.75 ± 1.32	19.4
	SER34	-1.32 ± 0.38	13.9		ALA158	-0.93 ± 0.49	16.9
	ILE35	0.52 ± 0.26	16.4		ILE159	-1.68 ± 0.75	13.7

	HIE36	-1.15 ± 0.34	20.1		ALA160	-2.76 ± 0.69	15.7
	THR37	-1.75 ± 0.27	21.6		ALA161	-0.02 ± 0.59	16.5
	ARG38	89.36 ± 1.04	21.9		GLY162	4.71 ± 1.16	12.8
	LYS42	58.44 ± 0.41	29.1		ILE163	1.79 ± 0.65	12.4
	SER55	0.29 ± 0.13	26.9		PHE164	0.78 ± 0.41	15.8
	GLY56	-1.02 ± 0.10	25.9		ASN165	6.23 ± 0.58	15.2
	PHE57	0.64 ± 0.07	25.8		ASP166	-146.17 ± 3.40	11.8
	HIE58	-0.09 ± 0.14	26.8		LEU167	1.38 ± 0.93	10.7
	TYR105	-0.97 ± 0.10	29.8		GLY168	-15.89 ± 5.33	6.9
	TYR107	0.00 ± 0.16	30.1		SER169	12.29 ± 3.66	7.6
	GLY139	0.38 ± 0.12	24.1		GLY170	-11.30 ± 2.50	10.6
	SER140	0.89 ± 0.40	20.4		SER171	8.54 ± 3.75	13.2
	ALA141	0.20 ± 0.19	20.6		ASN172	-12.74 ± 3.09	14.5
	SER142	-0.25 ± 0.57	24.2		ILE173	5.17 ± 0.76	14.3
	ALA143	0.80 ± 0.22	23.5		ASP174	-135.07 ± 4.79	14.8
	MET144	0.05 ± 0.21	22.4		LEU175	-0.80 ± 0.51	17.0
	PHE179	-1.85 ± 0.11	25.2		CYS176	-0.21 ± 0.64	19.0
	ILE180	-2.60 ± 0.09	23.8		VAL177	-0.69 ± 0.21	22.2
	SER181	-2.67 ± 0.31	22.5		ILE178	-0.70 ± 0.14	24.5
	ALA182	-2.80 ± 0.21	20.9		LEU183	1.65 ± 0.11	26.1
	ALA183	-3.51 ± 0.20	19.3		ASP184	-64.50 ± 0.79	24.6
	GLU184	-97.49 ± 1.48	17.8		PHE185	2.75 ± 0.24	21.4
	ARG185	98.01 ± 1.52	15.9		LEU186	-3.40 ± 0.27	20.6
	ASP186	-81.51 ± 1.00	15.5		ARG187	125.27 ± 2.92	18.6
	VAL187	2.17 ± 0.52	13.4		PRO188	4.88 ± 0.34	18.1
	TYR188	0.08 ± 0.26	16.0		TYR189	3.70 ± 0.28	19.8
	THR189	-0.78 ± 0.31	18.7		THR190	-2.81 ± 0.52	19.3
	GLY190	1.48 ± 0.19	19.9		VAL191	3.15 ± 0.23	18.7
	ASP191	-76.88 ± 0.83	21.5		PRO192	5.44 ± 0.52	18.3
	ALA192	-0.60 ± 0.16	25.2		ASN193	-1.95 ± 0.73	20.0
	LEU210	0.63 ± 0.18	22.9		LYS194	72.78 ± 1.11	21.1
	ARG211	126.01 ± 2.80	19.3		LYS195	76.01 ± 1.06	23.6
	LYS212	74.76 ± 1.14	18.9				
	ASP213	-237.55 ± 4.08	15.1				
β7'	TRP107	-0.23 ± 0.09	30.3				
	TYR124	0.34 ± 0.22	29.1				
	ASH126	0.90 ± 0.12	28.4				
	LEU128	0.59 ± 0.11	29.4				
	VAL130	0.55 ± 0.14	27.7				
	TYR132	0.58 ± 0.23	27.8				
	THR139	-0.36 ± 0.27	23.3				
	GLY140	-1.32 ± 0.26	21.0				
	TYR141	-0.22 ± 0.19	18.5				
	GLY142	-1.15 ± 0.18	21.1				
	ALA143	-0.98 ± 0.25	19.5				
	TYR144	-2.48 ± 0.39	16.9				
	LEU145	-0.33 ± 0.28	19.4				
	ALA146	0.51 ± 0.21	22.7				
	GLN147	2.41 ± 0.27	21.9				
	PRO148	1.08 ± 0.17	20.6				
	ARG151	72.82 ± 0.77	23.9				
	ARG179	64.59 ± 0.65	21.5				
	ASP180	-54.46 ± 0.50	22.2				

4.3 Electrostatic potential (V_{elec}) in β1-subunit.

Table S4. Electrostatic potential (V_{elec}) generated in the active site of β1 subunit by each residue located within 20 Å.

Subunit	Residue	V_{elec} (kJ/mol·e$^-$)	Distance (Å)	Catalytic Subunit	Residue	V_{elec} (kJ/mol·e$^-$)	Distance (Å)
α1	LYS102	52.85 ± 0.54	27.4	β1	THR2	-3.33 ± 3.17	7.9
	TYR103	0.03 ± 0.19	25.1		ILE3	-0.10 ± 0.97	10.1
	LYS104	42.74 ± 0.30	26.3		MET4	-4.64 ± 0.56	13.9
	TYR105	-0.75 ± 0.19	26.2		ALA5	-1.17 ± 0.23	17.3
	GLY106	0.40 ± 0.15	24.9		VAL6	-1.03 ± 0.24	20.9
					VAL12	0.95 ± 0.21	23.6
β2	ILE3	-1.19 ± 0.11	24.9		VAL13	0.61 ± 0.23	21.3
	ALA4	1.29 ± 0.14	24.3		LEU14	2.97 ± 0.38	17.5
	GLY5	-1.35 ± 0.14	25.3		GLY15	2.21 ± 0.83	14.7
	VAL6	1.04 ± 0.11	25.9		ALA16	17.35 ± 1.77	11.3
	VAL7	-0.05 ± 0.12	27.3		ASP17	-383.37 ± 24.01	10.3
	CYS43	0.85 ± 0.37	27.2		SER18	35.39 ± 5.33	9.9
	CYS44	-1.35 ± 0.27	26.6		ARG19	135.84 ± 15.60	7.8
	GLY45	0.76 ± 0.17	24.6		THR20	-21.52 ± 2.80	6.6
	ALA46	0.67 ± 0.22	23.7		THR21	-10.79 ± 1.64	7.2
	GLY47	1.27 ± 0.11	22.4		THR22	-0.25 ± 1.36	9.2
	THR48	-1.13 ± 0.11	24.9		GLY23	-6.62 ± 0.47	10.5
	THR55	0.26 ± 0.18	26.8		SER24	-4.87 ± 1.05	10.9
	ILE59	0.69 ± 0.08	28.4		TYR25	-5.67 ± 0.93	12.3
	VAL76	-0.28 ± 0.16	26.6		ILE26	6.10 ± 0.70	11.0
	VAL77	-0.78 ± 0.13	24.5		ALA27	6.02 ± 0.79	11.7
	THR78	-1.45 ± 0.25	26.5		ASN28	-10.28 ± 2.20	11.6
	ALA79	-0.22 ± 0.13	25.7		ARG29	100.24 ± 2.52	12.3
	ASN80	-0.11 ± 0.48	21.9		VAL30	6.31 ± 1.13	13.4
	ARG81	64.97 ± 0.62	21.8		THR31	11.91 ± 5.67	11.0
	MET82	-0.27 ± 0.21	23.8		ASP32	-134.03 ± 4.02	12.3
	LEU83	0.39 ± 0.18	21.8		LYN34	-12.59 ± 1.82	13.1
	LYS84	84.46 ± 1.29	18.3		LEU35	-5.12 ± 1.15	15.2
	GLN85	1.71 ± 0.26	20.1		THR36	-4.06 ± 0.70	19.0
	MET86	0.49 ± 0.19	22.4		PRO37	-2.05 ± 0.43	20.5
	LEU87	1.56 ± 0.22	19.7		ILE38	0.62 ± 0.20	24.2
	PHE88	0.70 ± 0.18	17.9		HIE39	-2.81 ± 0.17	26.5
	ARG89	46.71 ± 0.42	21.4		ASP40	-54.11 ± 0.35	25.4
	TYR90	0.41 ± 0.15	21.3		ARG41	50.78 ± 0.30	22.1
	GLN91	-0.65 ± 0.42	17.8		ILE42	0.41 ± 0.22	18.4
	GLY92	0.90 ± 0.17	18.5		PHE43	2.80 ± 0.34	15.0
	TYR93	0.13 ± 0.15	22.1		CYS44	-1.22 ± 1.04	11.5
	ILE94	-1.21 ± 0.15	21.9		CYS45	12.05 ± 1.52	8.3
	GLY95	-0.58 ± 0.20	21.2		ARG46	133.95 ± 3.86	5.1
	ALA96	-1.83 ± 0.15	21.7		SER47	16.97 ± 2.57	4.6
	ALA97	1.77 ± 0.12	19.9		GLY48	-7.12 ± 1.02	6.7
	LEU98	-2.16 ± 0.12	21.3		SER49	-3.41 ± 0.94	7.0
	VAL99	1.30 ± 0.09	21.8		ALA50	1.91 ± 0.74	10.8
	LEU100	-1.35 ± 0.12	23.7		ALA51	0.34 ± 0.50	11.5
	GLY101	0.44 ± 0.08	25.9		ASP52	-85.31 ± 1.48	10.1
	HID109	0.03 ± 0.23	25.3		THR53	1.00 ± 1.18	12.0
	LEU110	-1.38 ± 0.16	23.0		GLN54	-2.34 ± 1.04	15.0
	TYR111	2.60 ± 1.05	19.8		ALA55	1.57 ± 0.24	15.2
	SER112	-1.24 ± 0.66	17.9		VAL56	3.16 ± 0.23	15.2
	ILE113	1.78 ± 0.19	16.6		ALA57	3.51 ± 0.23	17.8
	TYR114	1.15 ± 0.75	14.9		ASP58	-68.01 ± 0.72	19.9
	PRO115	1.11 ± 0.16	16.5		ALA59	1.69 ± 0.12	20.1
	HID116	2.56 ± 0.69	14.7		VAL60	2.31 ± 0.15	20.9
	GLY117	0.22 ± 0.47	15.2		THR61	0.95 ± 0.25	23.9
	SER118	4.50 ± 1.43	12.3		TYR62	0.69 ± 0.14	25.0
	THR119	-5.43 ± 0.75	14.3		GLN63	-0.47 ± 0.12	25.2
	ASP120	-136.44 ± 2.70	14.4		LEU64	1.65 ± 0.11	26.9
	LYS121	74.90 ± 1.56	17.6		GLY76	1.14 ± 0.08	24.6

	LEU122	2.14 ± 0.36	18.5		VAL78	-0.92 ± 0.11	25.6
	PRO123	2.04 ± 0.12	22.1		THR79	-0.25 ± 0.14	22.2
	TYR124	-3.10 ± 0.33	21.9		ALA80	-0.33 ± 0.23	20.4
	VAL125	2.17 ± 0.13	20.0		ALA81	-0.97 ± 0.16	22.6
	THR126	-3.92 ± 0.28	20.3		SER82	-0.23 ± 0.26	21.6
	MET127	0.82 ± 0.35	19.8		LEU83	0.08 ± 0.17	17.8
	GLY128	-1.77 ± 0.17	21.1		PHE84	-0.02 ± 0.38	19.1
	SER129	-1.20 ± 0.27	21.5		LYS85	52.40 ± 0.40	21.3
	GLY130	-0.20 ± 0.19	22.9		GLU86	-51.90 ± 0.31	18.7
	SER131	0.80 ± 0.41	20.6		MET87	1.57 ± 0.37	16.1
	LEU132	-0.58 ± 0.27	18.5		CYS88	-1.36 ± 0.32	19.2
	ALA133	-0.88 ± 0.24	22.0		TYR89	-0.30 ± 0.21	20.7
	ALA134	0.40 ± 0.16	23.1		ARG90	54.20 ± 0.42	18.1
	MET135	2.52 ± 0.43	20.2		TYR91	-0.91 ± 0.27	16.9
	ALA136	-0.12 ± 0.22	20.8		ARG92	56.40 ± 0.47	18.0
	VAL137	0.34 ± 0.17	24.6		GLU93	-56.59 ± 0.40	16.0
	PHE138	1.27 ± 0.20	24.1		ASP94	-67.07 ± 1.09	12.7
	GLU139	-86.76 ± 1.02	22.8		LEU95	2.34 ± 0.17	9.7
	PHE142	0.60 ± 0.20	26.8		MET96	-4.86 ± 0.73	8.9
					ALA97	4.54 ± 0.40	9.8
β7	THR1	49.73 ± 0.29	22.9		GLY98	-5.21 ± 0.91	11.5
	ASN3	0.51 ± 0.28	20.5		ILE99	1.87 ± 0.51	13.8
	PRO4	-1.07 ± 0.13	19.9		ILE100	-2.19 ± 0.58	17.0
	MET5	-1.93 ± 0.50	16.6		ILE101	-0.29 ± 0.47	19.6
	VAL6	0.54 ± 0.12	18.1		ALA102	-1.10 ± 0.30	22.9
	THR7	-0.29 ± 0.13	21.7		GLY110	-0.44 ± 0.15	24.7
	SER29	0.26 ± 0.22	20.3		GLN111	-0.51 ± 0.26	21.1
	TYR30	-0.48 ± 1.19	17.9		VAL112	1.96 ± 0.22	19.6
	GLY31	-0.37 ± 0.30	17.4		TYR113	-1.89 ± 0.27	16.4
	SER32	1.66 ± 0.17	20.4		SER114	2.01 ± 0.46	15.4
	LEU33	0.71 ± 0.22	18.9		VAL115	-2.48 ± 0.27	13.5
	ALA34	-0.42 ± 0.18	21.3		PRO116	1.59 ± 0.48	12.9
	ARG35	93.18 ± 1.41	18.5		MET117	3.09 ± 0.67	16.3
	PHE36	0.68 ± 0.14	21.0		GLY118	1.42 ± 0.16	17.4
	ASP56	-55.14 ± 0.42	22.9		GLY119	2.29 ± 0.17	18.6
	TYR57	1.77 ± 0.13	22.9		MET120	-1.86 ± 0.35	19.8
	ALA58	0.81 ± 0.12	21.2		MET121	1.02 ± 0.29	21.2
	GLN61	-1.08 ± 0.15	25.0		VAL122	-1.71 ± 0.12	22.8
					ARG123	51.89 ± 0.35	22.4
β6'	ARG1	105.14 ± 0.69	25.8		GLN125	-3.18 ± 0.31	20.6
	PHE2	-0.20 ± 0.08	26.2		PHE126	2.15 ± 0.16	17.1
	PHE33	0.21 ± 0.13	25.9		ALA127	-1.06 ± 0.39	13.9
	SER34	0.82 ± 0.18	26.7		ILE128	4.81 ± 0.39	10.7
					GLY129	-3.30 ± 0.99	7.2
β7'	GLN2	-0.17 ± 0.12	24.2		GLY130	10.57 ± 0.84	6.6
	ASN3	-1.03 ± 0.16	23.1		SER131	21.62 ± 2.18	9.9
	PRO4	-0.66 ± 0.21	22.2		GLY132	7.95 ± 0.87	11.9
	MET5	-2.54 ± 0.14	21.4		SER133	8.34 ± 0.67	13.8
	VAL6	1.41 ± 0.08	20.6		SER134	5.75 ± 0.36	15.3
	THR7	-2.50 ± 0.29	21.1		TYR135	1.41 ± 0.54	16.8
	GLY8	0.34 ± 0.25	19.9		ILE136	2.17 ± 0.35	18.6
	THR9	3.52 ± 0.23	22.2		TYR137	2.52 ± 0.40	21.8
	SER10	0.21 ± 0.22	23.4		GLY138	1.33 ± 0.18	21.8
	ASP25	-67.56 ± 0.86	23.9		TYR139	1.40 ± 0.23	21.1
	MET26	0.57 ± 0.19	20.4		VAL152	1.89 ± 0.17	24.1
	LEU27	2.41 ± 0.26	19.9		CYS153	-1.82 ± 0.20	22.8
	GLY28	-0.24 ± 0.41	16.6		LEU155	-2.93 ± 0.18	22.3
	SER29	3.75 ± 0.35	16.7		PHE156	-2.12 ± 0.25	18.9
	TYR30	1.40 ± 0.92	13.7		THR157	-3.41 ± 0.55	19.1
	GLY31	-0.48 ± 0.57	10.9		ALA158	-3.56 ± 0.46	20.1
	SER32	1.35 ± 0.86	13.3		ASN159	-3.76 ± 0.50	17.4
	LEU33	1.07 ± 0.40	15.3		ALA160	-1.55 ± 0.51	14.5
	ALA34	1.20 ± 0.31	18.5		LEU161	-2.28 ± 0.93	16.4
	ARG35	62.42 ± 0.55	21.0		ALA162	-2.56 ± 0.82	16.7

	PHE36	-1.76 ± 0.31	21.5		LEU163	-0.73 ± 0.53	13.0
	ARG37	71.87 ± 0.78	26.4		ALA164	2.35 ± 0.81	12.6
	ILE39	-0.71 ± 0.10	27.9		MET165	2.70 ± 1.83	15.3
	ARG41	59.67 ± 0.49	24.5		GLU166	-91.10 ± 1.48	14.1
	SER54	2.12 ± 0.16	23.2		ARG167	86.33 ± 1.55	10.7
	GLY55	-0.08 ± 0.19	24.3		ASP168	-140.93 ± 3.35	10.1
	ASP56	-49.07 ± 0.37	26.2		GLY169	-5.95 ± 1.24	6.3
	TYR57	0.80 ± 0.13	24.7		SER170	-27.51 ± 3.20	8.0
	TRP107	0.75 ± 0.18	27.6		SER171	30.01 ± 3.72	11.0
	THR109	-0.80 ± 0.18	25.5		GLY172	-13.69 ± 2.17	13.5
	THR139	-1.19 ± 0.18	21.8		GLY173	3.11 ± 0.75	15.0
	GLY140	1.30 ± 0.13	19.4		VAL174	-12.38 ± 1.40	14.9
	TYR141	1.19 ± 0.43	22.4		ILE175	5.52 ± 0.53	15.1
	GLY142	0.03 ± 0.17	24.4		ARG176	135.51 ± 2.68	17.7
	ALA143	0.88 ± 0.10	22.9		LEU177	-1.13 ± 0.50	19.9
	TYR144	2.00 ± 0.27	21.0		ALA178	-1.73 ± 0.32	22.7
	LEU145	0.86 ± 0.21	24.0		ALA179	-0.58 ± 0.33	24.5
	ALA146	0.49 ± 0.10	25.4		ILE184	-0.46 ± 0.17	25.7
	CYS172	-1.73 ± 0.16	23.6		VAL185	1.67 ± 0.20	24.8
	MET173	-3.51 ± 0.19	22.3		GLU186	-61.69 ± 0.50	21.9
	ARG174	59.99 ± 0.43	21.0		ARG187	109.05 ± 1.63	21.6
	VAL175	-3.48 ± 0.27	19.1		GLN188	-2.51 ± 0.50	19.5
	LEU176	-4.15 ± 0.23	17.7		VAL189	4.37 ± 0.25	19.8
	TYR177	-5.52 ± 0.63	16.5		LEU190	-4.67 ± 0.35	19.7
	TYR178	-5.95 ± 0.82	15.2		LEU191	1.16 ± 0.46	18.7
	ARG179	104.05 ± 1.67	14.5		GLY192	1.84 ± 0.40	22.5
	ASP180	-89.97 ± 1.05	11.5		ASP193	-67.42 ± 0.57	23.1
	ALA181	-0.53 ± 0.57	14.3		GLN194	-0.12 ± 0.34	20.5
	ARG182	112.48 ± 2.21	17.0		ILE195	3.93 ± 0.25	22.4
	SER183	-4.04 ± 0.74	18.8		PRO197	-2.72 ± 0.22	23.4
	TYR184	2.07 ± 0.44	21.5				
	ASN185	3.89 ± 0.35	23.8				
	ARG186	55.96 ± 0.42	23.1				
	THR207	0.88 ± 0.46	22.9				
	ASN208	-3.14 ± 0.45	20.6				
	TRP209	-4.76 ± 0.39	22.7				
	ASP210	-71.26 ± 0.65	20.5				
	ILE211	-0.18 ± 0.50	18.4				
	ALA212	-1.04 ± 0.36	21.5				
	HID213	0.90 ± 0.54	22.6				
	MET214	-1.34 ± 0.45	20.8				
	ILE215	2.87 ± 0.23	22.6				
	SER216	-1.27 ± 0.37	24.9				
	GLY217	0.98 ± 0.27	24.0				
	PHE218	-1.60 ± 0.22	21.8				
	GLU219	-152.71 ± 1.43	29.3				
β1'	MET95	0.72 ± 0.11	26.6				
	MET116	0.19 ± 0.13	21.6				
	SER130	-1.48 ± 0.32	22.4				
	GLY131	-1.55 ± 0.21	22.7				
	SER132	-1.37 ± 0.18	18.9				
	SER133	-0.50 ± 0.39	18.8				
	TYR134	0.27 ± 0.38	21.4				
	ILE135	0.30 ± 0.21	20.1				
	TYR136	1.36 ± 0.26	19.5				
	GLY137	1.20 ± 0.23	23.7				
	ASP140	-65.45 ± 0.91	22.2				

5. Electrostatic potential (V_{elec}) generated per amino acid residue on Cl- leaving in the E-PC complex.

Table S5. Electrostatic potential (V_{elec}) generated in the active site of β1 subunit by each residue located within 20 Å.

Subunit	Residue	V_{elec} (kJ/mol·e^{-})	Distance (Å)	Catalytic Subunit	Residue	V_{elec} (kJ/mol·e^{-})	Distance (Å)
α1	LYS102	26.97 ± 0.08	33.7	β1	THR2	-0.02 ± 0.09	11.8
	TYR103	-0.31 ± 0.04	31.2		ILE3	-0.29 ± 0.04	15.2
	LYS104	28.42 ± 0.08	31.9		MET4	-0.14 ± 0.06	18.7
	TYR105	-0.22 ± 0.05	32.1		ALA5	-0.30 ± 0.03	22.4
	GLY106	0.20 ± 0.03	31.1		VAL6	-0.01 ± 0.03	25.7
					VAL12	0.28 ± 0.03	29.2
β2	ILE3	-0.37 ± 0.04	25.7		VAL13	-0.15 ± 0.03	26.7
	ALA4	0.00 ± 0.05	25.8		LEU14	0.40 ± 0.03	23.0
	GLY5	-0.33 ± 0.03	27.6		GLY15	-0.19 ± 0.03	20.0
	VAL6	0.04 ± 0.03	28.6		ALA16	0.51 ± 0.03	16.3
	VAL7	-0.03 ± 0.03	30.6		ASP17	-37.14 ± 0.15	14.1
	CYS43	0.22 ± 0.06	30.2		SER18	0.24 ± 0.10	14.3
	CYS44	0.00 ± 0.11	28.8		ARG19	38.09 ± 0.22	11.0
	GLY45	0.42 ± 0.04	26.3		THR20	0.58 ± 0.12	9.8
	ALA46	0.40 ± 0.06	24.5		THR21	0.08 ± 0.23	7.6
	GLY47	0.84 ± 0.09	22.8		THR22	1.19 ± 0.20	8.5
	THR48	0.36 ± 0.10	25.1		GLY23	0.56 ± 0.18	6.6
	THR55	0.35 ± 0.06	29.6		SER24	0.11 ± 0.20	7.2
	ILE59	0.37 ± 0.02	32.3		TYR25	-0.30 ± 0.08	10.4
	VAL76	-0.37 ± 0.02	31.6		ILE26	0.31 ± 0.06	10.8
	VAL77	-0.47 ± 0.03	30.0		ALA27	0.69 ± 0.05	12.9
	THR78	-0.47 ± 0.04	31.9		ASN28	-0.10 ± 0.07	14.2
	ALA79	-0.44 ± 0.03	30.4		ARG29	31.3 ± 0.09	14.7
	ASN80	-0.91 ± 0.07	26.8		VAL30	0.27 ± 0.04	17.1
	ARG81	30.04 ± 0.12	27.2		THR31	0.66 ± 0.08	15.8
	MET82	-0.54 ± 0.06	28.8		ASP32	-31.42 ± 0.12	17.9
	LEU83	-0.6 ± 0.03	26.2		LEU34	-0.32 ± 0.05	19.7
	LYS84	33.72 ± 0.15	23.1		THR35	-0.45 ± 0.06	22.2
	GLN85	-0.49 ± 0.10	25.0		PRO36	-0.44 ± 0.05	25.9
	MET86	-0.68 ± 0.07	26.6		ILE37	0.02 ± 0.03	27.2
	LEU87	-0.77 ± 0.07	23.3		HIE38	-0.22 ± 0.03	30.9
	PHE88	-0.82 ± 0.10	21.9		ASP39	-24.92 ± 0.06	33.3
	ARG89	38.17 ± 0.19	25.1		ARG40	23.85 ± 0.04	32.0
	TYR90	-0.07 ± 0.11	23.9		ILE41	0.14 ± 0.04	28.9
	GLN91	-0.97 ± 0.37	20.1		PHE42	0.05 ± 0.03	25.2
	GLY92	-1.08 ± 0.18	19.4		CYS43	0.28 ± 0.11	21.7
	TYR93	-0.03 ± 0.21	23.0		CYS44	0.17 ± 0.09	18.2
	ILE94	-0.75 ± 0.23	23.4		ARG45	35.91 ± 0.17	15.2
	GLY95	0.06 ± 0.10	22.1		SER46	0.38 ± 0.12	11.8
	ALA96	-0.75 ± 0.13	23.2		GLY47	0.98 ± 0.09	9.8
	ALA97	0.03 ± 0.05	21.6		SER48	0.75 ± 0.13	10.8
	LEU98	-0.39 ± 0.05	23.7		ALA49	0.70 ± 0.07	12.3
	VAL99	-0.12 ± 0.05	24.7		ALA50	0.96 ± 0.07	15.5
	LEU100	-0.13 ± 0.04	27.3		ASP51	-48.04 ± 0.28	16.4
	GLY101	-0.15 ± 0.02	29.7		THR52	0.97 ± 0.07	15.9
	HID109	-0.04 ± 0.04	30.0		GLN53	1.33 ± 0.10	18.2
	LEU110	0.36 ± 0.10	27.1		ALA54	0.75 ± 0.06	20.7
	TYR111	-0.08 ± 0.08	23.9		VAL55	0.69 ± 0.04	21.1
	SER112	0.05 ± 0.06	21.3		ALA56	0.46 ± 0.04	21.7
	ILE113	0.33 ± 0.09	20.1		ASP57	-33.37 ± 0.14	24.0
	TYR114	0.36 ± 0.10	17.5		ALA58	0.61 ± 0.04	26.0
	PRO115	0.47 ± 0.15	18.7		VAL59	0.49 ± 0.03	26.4
	HID116	-0.48 ± 0.13	17.7		THR60	0.56 ± 0.05	27.5
	GLY117	0.47 ± 0.05	19.4		TYR61	0.66 ± 0.19	30.2
	SER118	0.26 ± 0.09	16.8		GLN62	-0.06 ± 0.05	31.3

	THR119	-0.57 ± 0.17	19.0		LEU63	0.34 ± 0.02	31.7
	ASP120	-36.27 ± 0.20	19.2		GLY64	0.37 ± 0.01	33.6
	LYS121	28.75 ± 0.15	22.4		VAL76	-0.40 ± 0.02	30.9
	LEU122	0.13 ± 0.05	22.4		THR78	-0.49 ± 0.04	31.7
	PRO123	0.12 ± 0.03	25.5		ALA79	-0.48 ± 0.02	28.5
	TYR124	-0.30 ± 0.04	24.5		ALA80	-0.57 ± 0.02	26.4
	VAL125	0.46 ± 0.03	22.3		SER81	-0.74 ± 0.06	28.3
	THR126	-0.36 ± 0.15	21.7		LEU82	-0.65 ± 0.03	27.5
	MET127	0.15 ± 0.06	20.7		PHE83	-0.66 ± 0.03	23.7
	GLY128	-0.18 ± 0.11	21.0		LYS84	35.06 ± 0.12	24.3
	SER129	-0.26 ± 0.08	20.2		GLU85	-38.08 ± 0.10	26.3
	GLY130	0.20 ± 0.07	21.4		MET86	-0.65 ± 0.10	23.8
	SER131	0.34 ± 0.10	20.2		CYS87	-1.06 ± 0.07	20.8
	LEU132	0.84 ± 0.06	17.5		TYR88	-1.38 ± 0.11	23.2
	ALA133	0.45 ± 0.05	20.6		ARG89	37.55 ± 0.18	24.7
	ALA134	0.52 ± 0.02	22.7		TYR90	-0.26 ± 0.12	21.5
	MET135	0.71 ± 0.08	20.4		ARG91	50.43 ± 0.26	19.7
	ALA136	0.50 ± 0.04	20.0		GLU92	-65.31 ± 0.46	19.6
	VAL137	0.40 ± 0.03	23.9		ASP93	-55.60 ± 0.52	18.3
	PHE138	0.45 ± 0.02	24.5		LEU94	-1.61 ± 0.23	15.7
	GLU139	-31.14 ± 0.12	23.2		MET95	0.91 ± 0.13	12.4
	PHE142	0.15 ± 0.03	28.3		ALA96	-0.54 ± 0.08	13.1
					GLY97	-0.26 ± 0.06	14.9
β7	THR1	54.94 ± 0.17	24.3		ILE98	-0.36 ± 0.07	17.4
	ASN3	-1.28 ± 0.34	20.5		ILE99	-0.08 ± 0.07	19.7
	PRO4	-1.57 ± 0.12	20.4		ILE100	-0.11 ± 0.04	23.2
	MET5	0.47 ± 0.12	17.6		ALA101	-0.19 ± 0.03	25.8
	VAL6	-0.50 ± 0.15	19.6		GLY102	-0.09 ± 0.03	29.2
	THR7	0.44 ± 0.18	22.7		GLN110	-0.20 ± 0.05	30.3
	SER29	-0.68 ± 0.08	20.9		VAL111	0.16 ± 0.04	26.5
	TYR30	1.09 ± 0.15	17.9		TYR112	0.20 ± 0.05	24.9
	GLY31	0.26 ± 0.15	16.1		SER113	-0.02 ± 0.07	21.4
	SER32	0.49 ± 0.12	18.7		VAL114	-0.11 ± 0.06	20.2
	LEU33	-0.36 ± 0.07	18.3		PRO115	0.26 ± 0.07	17.4
	ALA34	0.42 ± 0.04	21.5		MET116	1.27 ± 0.19	16.1
	ARG35	44.02 ± 0.21	19.7		GLY117	0.19 ± 0.12	19.3
	PHE36	-0.21 ± 0.05	23.0		GLY118	0.17 ± 0.09	21.5
	ASP56	-48.40 ± 0.18	24.2		MET119	-0.38 ± 0.07	22.5
	TYR57	0.55 ± 0.07	25.1		MET120	-0.13 ± 0.08	24.3
	ALA58	0.93 ± 0.05	24.1		VAL121	-0.17 ± 0.05	25.4
	GLN61	-0.03 ± 0.07	28.6		ARG122	27.73 ± 0.09	27.3
					GLN123	-0.27 ± 0.09	26.7
β6'	ARG1	152.89 ± 0.94	16.2		PHE125	-0.04 ± 0.03	24.8
	PHE2	-2.43 ± 0.16	19.6		ALA126	0.39 ± 0.04	21.3
	PHE33	0.66 ± 0.08	22.7		ILE127	-0.09 ± 0.05	17.6
	SER34	-0.75 ± 0.18	22.2		GLY128	0.46 ± 0.08	14.5
					GLY129	0.47 ± 0.12	10.7
β7'	GLN2	-3.45 ± 0.61	20.1		SER130	-0.60 ± 0.15	7.8
	ASN3	0.65 ± 0.82	17.5		GLY131	-0.01 ± 0.09	10.1
	PRO4	-1.16 ± 0.13	16.5		SER132	0.44 ± 0.09	13.4
	MET5	-0.82 ± 0.16	15.6		SER133	0.38 ± 0.16	13.7
	VAL6	-0.46 ± 0.14	15.2		TYR134	0.47 ± 0.08	14.6
	THR7	0.44 ± 0.31	14.4		ILE135	0.45 ± 0.02	17.3
	GLY8	-0.89 ± 0.09	15.6		TYR136	0.83 ± 0.09	19.2
	THR9	-0.30 ± 0.12	14.5		GLY137	0.44 ± 0.04	21.8
	SER10	-0.09 ± 0.09	17.3		TYR138	0.39 ± 0.03	22.5
	ASP25	-37.39 ± 0.12	19.5		VAL139	0.56 ± 0.02	22.9
	MET26	0.03 ± 0.07	19.7		CYS152	-0.37 ± 0.02	28.3
	LEU27	0.02 ± 0.09	16.1		LEU153	-0.46 ± 0.02	27.0
	GLY28	0.35 ± 0.10	14.7		PHE155	-0.29 ± 0.03	25.5
	SER29	-0.71 ± 0.14	11.1		THR156	-0.57 ± 0.03	22.6
	TYR30	-0.69 ± 0.25	10.9		ALA157	-0.43 ± 0.04	22.3
	GLY31	0.07 ± 0.17	7.4		ASN158	-0.29 ± 0.05	22.3
	SER32	-0.08 ± 0.09	5.6		ALA159	-0.37 ± 0.04	19.6

	LEU33	-0.37 ± 0.07	9.1		LEU160	-0.49 ± 0.03	17.1
	ALA34	0.39 ± 0.05	11.5		ALA161	-0.44 ± 0.06	18.0
	ARG35	45.71 ± 0.22	14.2		LEU162	-0.42 ± 0.04	17.3
	PHE36	-0.45 ± 0.05	16.8		ALA163	-0.48 ± 0.04	13.8
	ARG37	32.81 ± 0.11	17.9		MET164	-0.31 ± 0.17	13.3
	ILE39	0.30 ± 0.07	21.6		GLU165	-33.38 ± 0.17	14.5
	ARG41	38.08 ± 0.15	22.6		ARG166	35.79 ± 0.13	12.6
	SER54	0.22 ± 0.16	18.6		ASP167	-44.31 ± 0.22	8.8
	GLY55	1.06 ± 0.10	16.9		GLY168	0.53 ± 0.14	7.4
	ASP56	-49.73 ± 0.24	17.7		SER169	-0.30 ± 0.08	5.5
	TYR57	0.41 ± 0.07	19.9		SER170	-0.61 ± 0.11	9.3
	TRP107	-0.51 ± 0.16	18.0		GLY171	-0.24 ± 0.07	12.4
	THR109	0.21 ± 0.14	21.5		GLY172	0.18 ± 0.04	15.6
	THR139	1.03 ± 0.08	20.4		VAL173	-0.49 ± 0.04	18.1
	GLY140	0.46 ± 0.15	16.6		ILE174	0.31 ± 0.02	18.8
	TYR141	0.01 ± 0.13	15.1		ARG175	30.12 ± 0.10	20.1
	GLY142	0.38 ± 0.05	18.4		LEU176	0.01 ± 0.05	22.9
	ALA143	0.31 ± 0.10	20.3		ALA177	-0.19 ± 0.03	25.6
	TYR144	0.30 ± 0.14	19.7		ALA178	0.00 ± 0.05	28.5
	LEU145	0.61 ± 0.05	18.5		ILE179	-0.05 ± 0.03	30.6
	ALA146	0.54 ± 0.02	21.3		VAL184	0.28 ± 0.03	32.0
	CYS172	-0.39 ± 0.05	23.2		GLU185	-24.23 ± 0.05	30.9
	MET173	-0.70 ± 0.05	21.1		ARG186	29.20 ± 0.09	27.7
	ARG174	27.79 ± 0.07	21.0		GLN187	-0.19 ± 0.06	26.8
	VAL175	-0.34 ± 0.04	19.7		VAL188	0.35 ± 0.02	24.1
	LEU176	-0.40 ± 0.04	16.8		LEU189	-0.32 ± 0.01	23.3
	TYR177	-0.13 ± 0.12	16.2		LEU190	0.03 ± 0.04	22.8
	TYR178	-0.25 ± 0.06	16.1		GLY191	0.05 ± 0.05	20.7
	ARG179	34.56 ± 0.12	14.0		ASP192	-26.19 ± 0.05	24.0
	ASP180	-41.48 ± 0.18	11.6		GLN193	-0.02 ± 0.04	25.2
	ALA181	0.10 ± 0.14	8.5		ILE194	0.34 ± 0.03	22.4
	ARG182	48.18 ± 0.23	10.0		PRO195	-0.44 ± 0.01	24.0
	SER183	0.00 ± 0.13	13.5		PHE197	-0.32 ± 0.03	23.0
	TYR184	-0.09 ± 0.11	16.1				
	ASN185	0.14 ± 0.06	19.5				
	ARG186	29.91 ± 0.06	21.2				
	THR207	0.37 ± 0.06	23.6				
	ASN208	-0.29 ± 0.06	24.5				
	TRP209	-0.33 ± 0.03	23.1				
	ASP210	-25.71 ± 0.05	25.9				
	ILE211	0.01 ± 0.07	23.8				
	ALA212	-0.11 ± 0.04	22.5				
	HID213	0.12 ± 0.07	26.0				
	MET214	-0.39 ± 0.06	26.9				
	ILE215	0.38 ± 0.02	25.8				
	SER216	-0.16 ± 0.04	27.4				
	GLY217	-0.03 ± 0.09	29.9				
	PHE218	-0.38 ± 0.03	29.5				
	GLU219	-56.95 ± 0.16	27.5				
β1'	MET95	0.03 ± 0.24	23.5				
	MET116	1.06 ± 0.13	20.6				
	SER130	0.73 ± 0.18	17.7				
	GLY131	0.06 ± 0.09	19.5				
	SER132	0.62 ± 0.06	19.9				
	SER133	1.12 ± 0.08	16.2				
	TYR134	0.57 ± 0.09	17.1				
	ILE135	0.44 ± 0.03	19.8				
	TYR136	0.47 ± 0.07	18.3				
	GLY137	0.47 ± 0.02	19.0				
	ASP140	-32.81 ± 0.07	22.3				

6. Stabilization of the final product of the reaction

6.1 Free energy surfaces (FESs) computed for β1-subunit

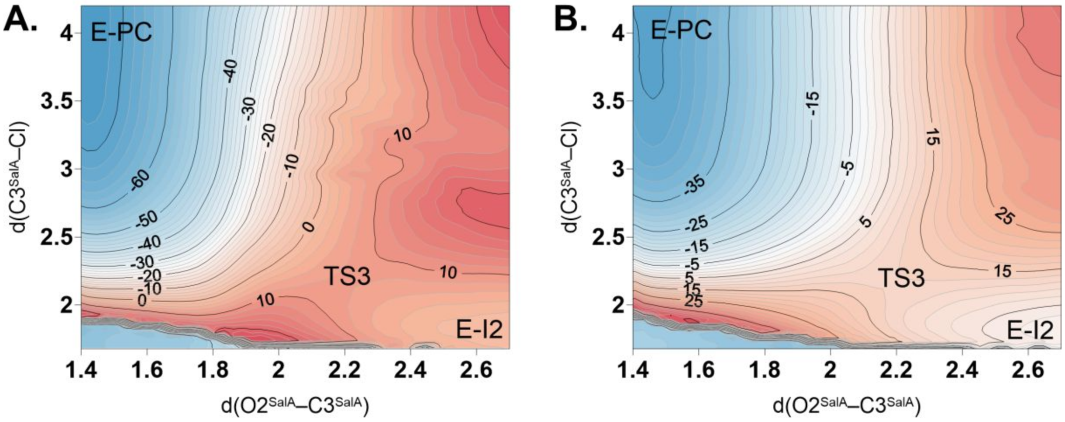

Figure S11. Free energy surfaces (FESs) computed at M06-2X:AM1/AMBER level for intramolecular cyclization step of inactivation of β1 subunit with SalA in two different model variants: **A.** electrostatically active Asp17 and water solvent(V_{elec}(D17&WAT)) and **B.** electrostatically active Asp17, water solvent and Asp167 (V_{elec}(D17&WAT&D167)). Values of energy are in kcal/mol.

6.2 Free energy surfaces (FESs) computed for β2-subunit

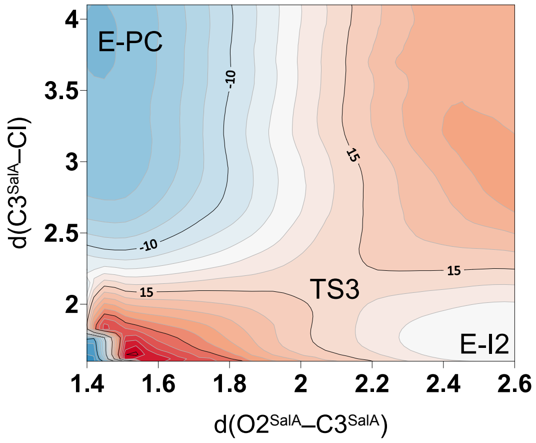

Figure S12. Free energy surfaces (FESs) computed at M06-2X:AM1/AMBER level for intramolecular cyclization step of inactivation of β2 subunit with SalA in electrostatically active Asp17 and water solvent(V_{elec}(D17&WAT)) variant. Values of energy are in kcal/mol.

6.3 Free energy surfaces (FESs) computed for β5-subunit

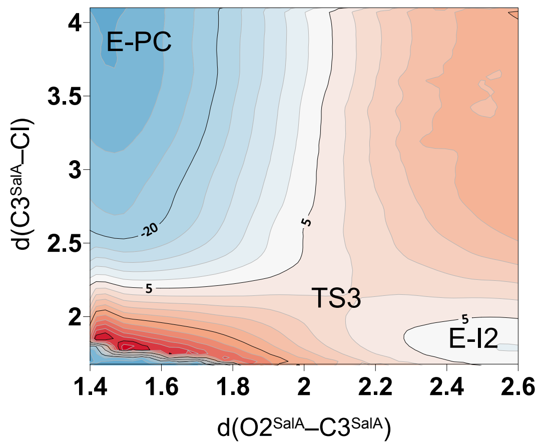

Figure S13. Free energy surfaces (FESs) computed at M06-2X:AM1/AMBER level for intramolecular cyclization step of inactivation of β5 subunit with SalA in electrostatically active Asp17 and water solvent(V_{elec}(D17&WAT)) variant. Values of energy are in kcal/mol.

7. Electrostatic field variations

7.1 Free energy surfaces (FESs) computed for β5-subunit at D17AA variants

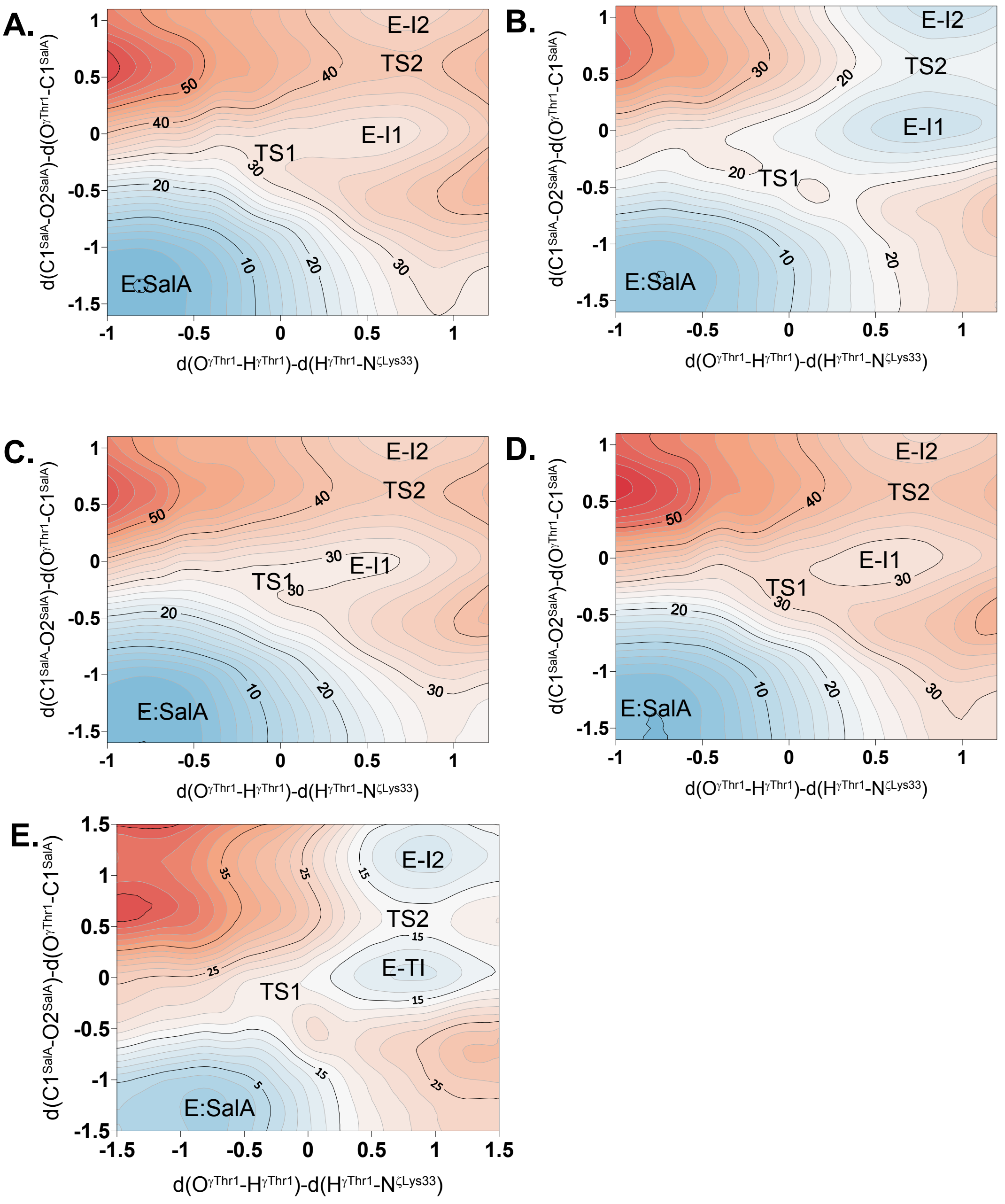

Figure S14. Free energy surfaces (FESs) computed at M06-2X:AM1/AMBER level for the first and second steps of SalA reactions in A. D17Ala, B. D17Cys, C. D17Leu, D. D17Ser and E. D17Glu β5 subunit variants. Values of energy are in kcal/mol.

7.2. Free energy surfaces (FESs) computed for β5-subunit at D17X variants

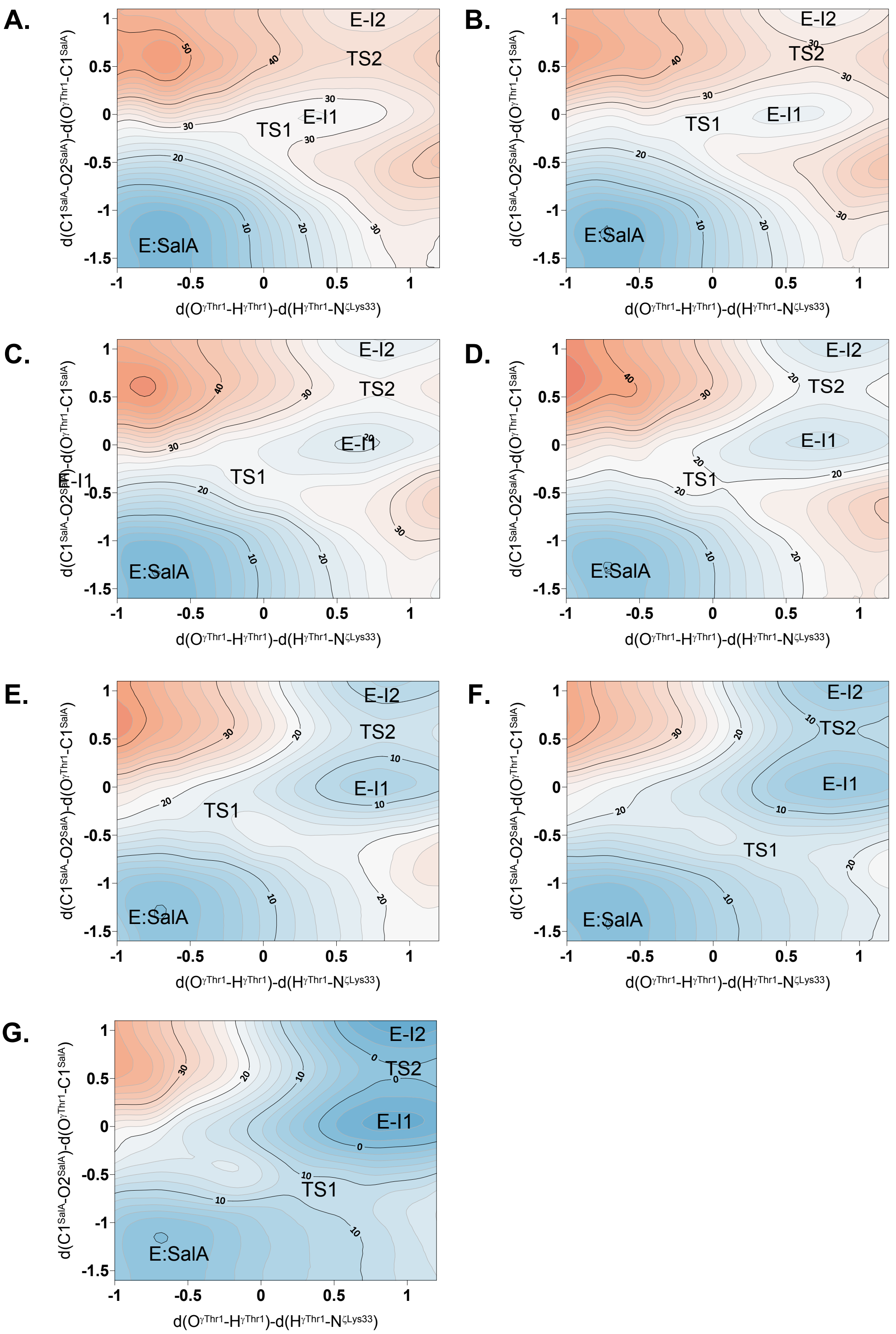

Figure S15. Free energy surfaces (FESs) computed at M06-2X:AM1/AMBER level for, the first and second step of SalA reactions as a function of the electrostatic potential, V_{elec} generated in the active site of β5 subunit by changing the contribution of point charges of Asp17 from A. 20%, B. 40%, C. 60%, D. 80%, E. 100%, F. 120% and G. 140 %. Values of energy are in kcal/mol.

8 Computational methods

8.1 Structure of the active site

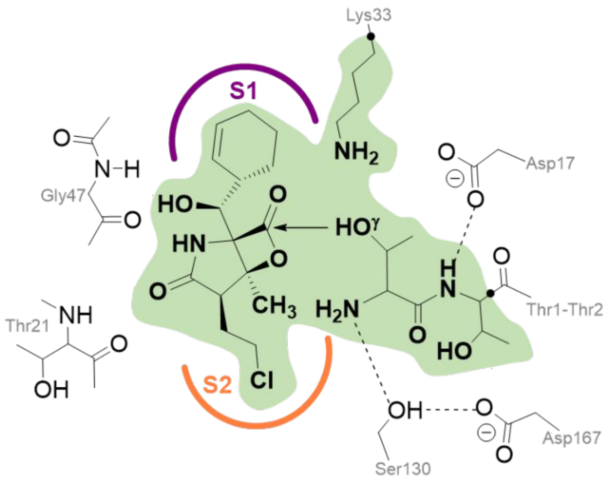

Figure S16. Structure of the active site of β5, β2, and β1 subunits of human 20S proteasome with bound SalA. Atoms included in the green area were described at the QM level of theory. Two black dots indicate the positions of link atoms.

8.2 Interactions diagrams of the active sites of each catalytic subunit

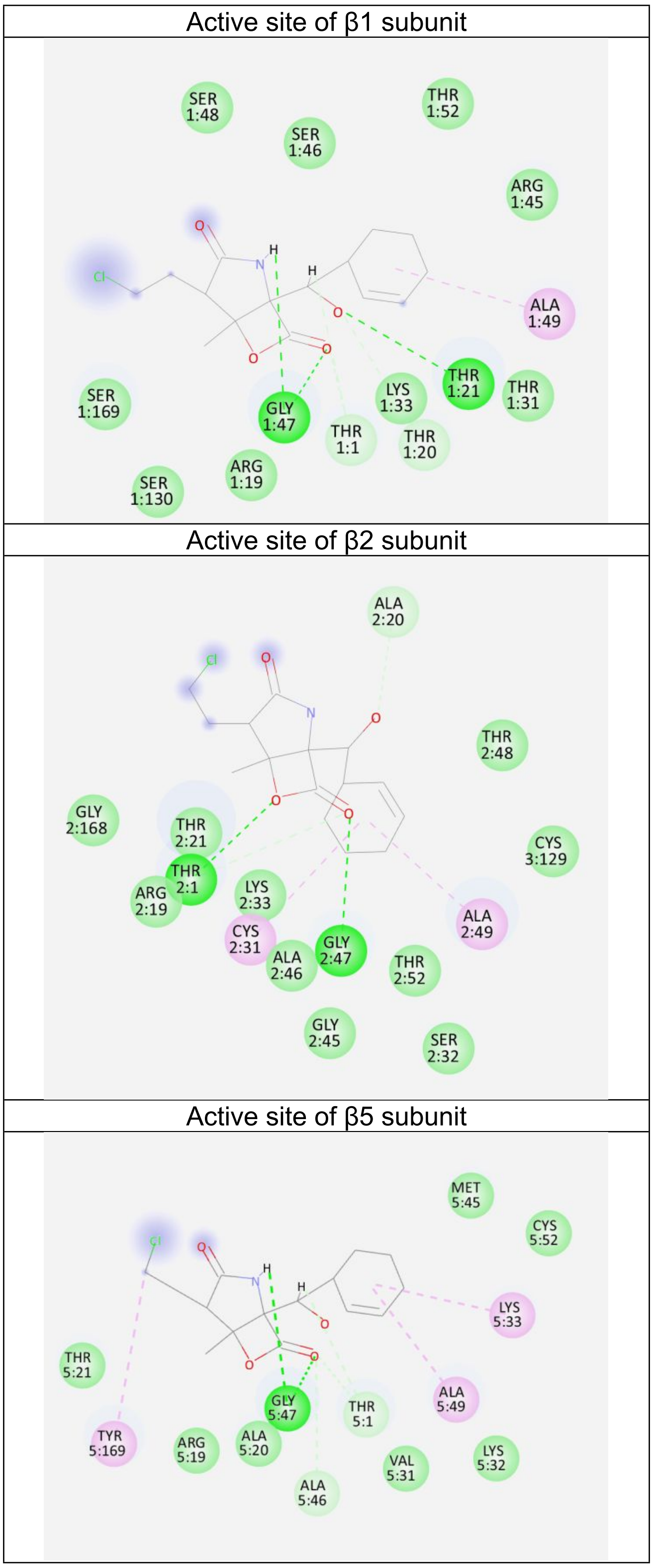

Figure S17. Ligand interaction diagram of SalA in the active site of β1, β2 and β5 subunits. Spheres highlighted in dark green correspond to conventional hydrogen bonds, in light green to van der Waals and carbon hydrogen bonds, and in pink to alkyl and pi-alkyl interactions. Diagrams were obtained using Discovery Studio Visualizer.

9 Key distances of optimized stationary points

9.1 Structures of optimized stationary points in V_{elec}(ON) conditions

Table S6. Key distances for optimized and characterized stationary points at M06-2X/MM along reaction progress in the active site of β5-subunit of 20S proteasome. Values are given in Å.

Distances	E:SalA	TS1	E-TI	TS2	E-I2	TS3	E-PC
$N^{\zeta Lys33} - H^{\gamma Thr1}$	1.67	1.17	1.03	1.03	1.03	1.03	1.03
$H^{\gamma Thr1} - O^{\gamma Thr1}$	1.02	1.41	2.14	2.20	2.23	2.28	2.29
$O^{\gamma Thr1} - C1^{SalA}$	2.48	2.12	1.45	1.40	1.34	1.35	1.33
$C1^{SalA} - O1^{SalA}$	1.20	1.21	1.26	1.23	1.21	1.21	1.21
$O1^{SalA} - H^{Gly47}$	1.87	1.90	1.84	1.86	1.89	1.93	1.92
$C1^{SalA} - O2^{SalA}$	1.36	1.40	1.57	1.86	2.58	2.73	2.69
$O2^{SalA} - C3^{SalA}$	2.98	3.07	3.02	2.83	2.51	2.34	1.45
$C3^{SalA} - Cl^{SalA}$	1.83	1.83	1.84	1.87	1.92	2.09	3.46
$O2^{SalA} - N^{Thr1}$	3.58	3.48	3.41	3.52	3.61	3.92	3.62
$H^{\gamma Thr1} - O2^{SalA}$	3.79	3.91	4.20	4.38	4.77	5.04	4.88
$H^{\gamma Thr1} - N^{Thr1}$	3.34	3.55	3.77	3.79	3.78	3.78	3.83

Table S7. Key distances for optimized and characterized stationary points at M06-2X/MM along reaction progress in the active site of β2-subunit of 20S proteasome. Values are given in Å.

Distances	E:SalA	TS1	E-TI	TS2	E-I2	TS3	E-PC
$N^{\zeta Lys33} - H^{\gamma Thr1}$	1.68	1.06	1.04	1.03	1.03	1.03	1.03
$H^{\gamma Thr1} - O^{\gamma Thr1}$	1.02	1.81	2.05	2.01	2.21	2.29	2.36
$O^{\gamma Thr1} - C1^{SalA}$	2.72	2.03	1.51	1.39	1.35	1.35	1.34
$C1^{SalA} - O1^{SalA}$	1.20	1.22	1.26	1.21	1.21	1.21	1.21
$O1^{SalA} - H^{Gly47}$	1.86	1.95	1.97	1.95	1.92	1.89	1.91
$C1^{SalA} - O2^{SalA}$	1.35	1.40	1.51	2.07	2.58	2.70	2.70
$O2^{SalA} - C3^{SalA}$	4.24	4.24	4.23	4.17	2.68	2.06	1.46
$C3^{SalA} - Cl^{SalA}$	1.80	1.80	1.81	1.81	1.83	2.26	3.19
$O2^{SalA} - N^{Thr1}$	3.22	3.01	2.93	2.75	3.12	3.39	3.52
$H^{\gamma Thr1} - O2^{SalA}$	3.96	4.27	4.21	4.28	4.88	5.16	5.21
$H^{\gamma Thr1} - N^{Thr1}$	3.32	3.63	3.74	3.51	4.01	4.06	4.20

Table S8. Key distances for optimized and characterized stationary points at M06-2X/MM along reaction progress in the active site of β1-subunit of 20S proteasome. Values are given in Å.

Distances	E:SalA	TS1	E-TI	TS2	E-I2	TS3	E-PC
$N^{\zeta Lys33} - H^{\gamma Thr1}$	1.84	1.18	1.03	1.02	1.02	1.02	1.02
$H^{\gamma Thr1} - O^{\gamma Thr1}$	0.99	1.38	2.55	2.55	2.42	2.50	2.57
$O^{\gamma Thr1} - C1^{SalA}$	2.69	1.92	1.48	1.36	1.35	1.35	1.34
$C1^{SalA} - O1^{SalA}$	1.20	1.23	1.28	1.22	1.21	1.21	1.21
$O1^{SalA} - H^{Gly47}$	1.91	1.97	1.95	1.81	1.78	1.82	1.85
$C1^{SalA} - O2^{SalA}$	1.34	1.40	1.49	2.17	2.38	2.47	2.53
$O2^{SalA} - C3^{SalA}$	4.30	4.34	4.31	3.39	2.78	2.10	1.47
$C3^{SalA} - Cl^{SalA}$	1.80	1.81	1.81	1.82	1.84	2.24	3.26
$O2^{SalA} - N^{Thr1}$	3.75	3.42	3.32	2.98	3.01	3.45	3.73
$H^{\gamma Thr1} - O2^{SalA}$	3.92	3.82	4.73	4.92	4.95	5.22	5.37
$H^{\gamma Thr1} - N^{Thr1}$	3.26	3.47	3.52	3.50	3.58	3.64	3.69

9.2 Structures of optimized stationary points in V_{elec}(OFF) conditions

Table S9. Key distances for optimized and characterized stationary points at M06-2X/MM along reaction progress in the active site of β5-subunit of 20S proteasome. Values are given in Å.

Distances	E:SalA	TS2	E-I2	TS3	E-PC
$N^{\zeta Lys33} - H^{\gamma Thr1}$	1.81	1.06	1.05	1.04	1.04
$H^{\gamma Thr1} - O^{\gamma Thr1}$	0.99	1.71	1.78	1.87	1.89
$O^{\gamma Thr1} - C1^{SalA}$	2.61	1.42	1.38	1.38	1.38
$C1^{SalA} - O1^{SalA}$	1.19	1.20	1.20	1.20	1.20
$O1^{SalA} - H^{Gly47}$	3.23	3.18	2.87	2.88	2.94
$C1^{SalA} - O2^{SalA}$	1.37	2.16	2.46	2.53	2.57
$O2^{SalA} - C3^{SalA}$	2.95	2.91	2.85	1.95	1.47
$C3^{SalA} - Cl^{SalA}$	1.80	1.81	1.82	2.35	3.18
$O2^{SalA} - N^{Thr1}$	3.62	3.03	2.95	3.19	3.30
$H^{\gamma Thr1} - O2^{SalA}$	3.75	3.96	4.11	4.30	4.34
$H^{\gamma Thr1} - N^{Thr1}$	3.44	3.84	3.73	3.78	3.81

9.3 Structures of optimized stationary points in (V_{elec}(D17) conditions

Table S10. Key distances for optimized and characterized stationary points at M06-2X/MM along reaction progress in the active site of β5-subunit of 20S proteasome. Values are given in Å.

Distances	E:SalA	TS1	E-TI	TS2	E-I2	TS3	E-PC
$N^{\zeta Lys33} - H^{\gamma Thr1}$	1.68	1.07	1.04	1.04	1.03	1.03	1.03
$H^{\gamma Thr1} - O^{\gamma Thr1}$	1.01	1.69	1.93	1.84	1.98	2.04	2.06
$O^{\gamma Thr1} - C1^{SalA}$	2.67	1.81	1.55	1.40	1.37	1.36	1.36
$C1^{SalA} - O1^{SalA}$	1.19	1.22	1.24	1.21	1.20	1.20	1.20
$O1^{SalA} - H^{Gly47}$	3.21	3.32	3.31	3.20	3.15	3.18	3.20
$C1^{SalA} - O2^{SalA}$	1.37	1.45	1.53	2.04	2.51	2.58	2.60
$O2^{SalA} - C3^{SalA}$	2.94	3.07	3.02	2.87	2.83	2.04	1.46
$C3^{SalA} - Cl^{SalA}$	1.80	1.81	1.81	1.82	1.83	2.29	3.35
$O2^{SalA} - N^{Thr1}$	3.73	3.39	3.30	3.11	3.12	3.43	3.45
$H^{\gamma Thr1} - O2^{SalA}$	3.84	3.98	4.04	4.06	4.34	4.53	4.51
$H^{\gamma Thr1} - N^{Thr1}$	3.45	3.75	3.81	3.90	3.78	3.83	3.84

Table S11. Key distances for optimized and characterized stationary points at M06-2X/MM along reaction progress in the active site of β2-subunit of 20S proteasome. Values are given in Å.

Distances	E:SalA	TS1	E-TI	TS2	E-I2	TS3	E-PC
$N^{\zeta Lys33} - H^{\gamma Thr1}$	1.67	1.09	1.04	1.04	1.03	1.03	1.03
$H^{\gamma Thr1} - O^{\gamma Thr1}$	1.01	1.60	1.88	1.92	1.95	2.06	2.08
$O^{\gamma Thr1} - C1^{SalA}$	2.53	1.86	1.53	1.38	1.37	1.36	1.36
$C1^{SalA} - O1^{SalA}$	1.19	1.22	1.25	1.21	1.20	1.20	1.20
$O1^{SalA} - H^{Gly47}$	2.45	2.51	2.51	2.29	2.35	2.36	2.36
$C1^{SalA} - O2^{SalA}$	1.36	1.43	1.51	2.18	2.43	2.54	2.48
$O2^{SalA} - C3^{SalA}$	4.25	4.27	4.24	4.19	2.69	2.03	1.44
$C3^{SalA} - Cl^{SalA}$	1.81	1.81	1.81	1.82	1.82	2.24	3.33
$O2^{SalA} - N^{Thr1}$	3.16	3.01	2.96	2.81	2.95	3.14	3.09
$H^{\gamma Thr1} - O2^{SalA}$	3.79	3.99	4.07	4.32	4.51	4.75	4.65
$H^{\gamma Thr1} - N^{Thr1}$	3.37	3.66	3.78	3.77	3.82	3.91	3.92

10. System modifications

Table S12. Point charges assigned to Asp17 atoms depending on their contribution to the electrostatic field.

Atoms	20%	40%	60%	80%	100%	120%	140%
**N**	-0.10326	-0.20652	-0.30978	-0.41304	-0.5163	-0.61956	-0.72282
**H**	0.05872	0.11744	0.17616	0.23488	0.2936	0.35232	0.41104
**CA**	0.00762	0.01524	0.02286	0.03048	0.0381	0.04572	0.05334
**HA**	0.0176	0.0352	0.0528	0.0704	0.0880	0.1056	0.1232
**CB**	-0.00606	-0.01212	-0.01818	-0.02424	-0.0303	-0.03636	-0.04242
**HB3**	-0.00244	-0.00488	-0.00732	-0.00976	-0.0122	-0.01464	-0.01708
**HB2**	-0.00244	-0.00488	-0.00732	-0.00976	-0.0122	-0.01464	-0.01708
**CG**	0.15988	0.31976	0.47964	0.63952	0.7994	0.95928	1.11916
**OD1**	-0.16028	-0.32056	-0.48084	-0.64112	-0.8014	-0.96168	-1.12196
**OD2**	-0.16028	-0.32056	-0.48084	-0.64112	-0.8014	-0.96168	-1.12196
**C**	0.10732	0.21464	0.32196	0.42928	0.5366	0.64392	0.75124
**O**	-0.11638	-0.23276	-0.34914	-0.46552	-0.5819	-0.69828	-0.81466

11 References

(1) Waterhouse, A. M.; Procter, J. B.; Martin, D. M. A.; Clamp, M.; Barton, G. J. Jalview Version 2--a multiple sequence alignment editor and analysis workbench. *Bioinformatics* **2009**, 25, 1189-1191.

(2) Holm, L.; Laiho, A.; Toronen, P.; Salgado, M. DALI shines a light on remote homologs: One hundred discoveries. *Protein Science* **2023**, 23, e4519.
